# Loss of *arap3* drives a cerebral cavernous malformation-like phenotype in zebrafish via Krit1 interaction and RhoA dysregulation

**DOI:** 10.1242/dev.205762

**Published:** 2026-09-21

**Authors:** Di Yang, Zewen Tao, Si Chen, Hongcheng Wang, Lingfei Luo, Jingying Chen

**Affiliations:** ^1^ Institute of Developmental Biology and Regenerative Medicine, Southwest University, Beibei 400715, Chongqing, China; ^2^ School of Life Sciences, Fudan University, Shanghai 200438, China

**Keywords:** Cerebral cavernous malformations, *Arap3*, Vascular development, Vascular homeostasis, RhoA activity, Zebrafish

## Abstract

Cerebral cavernous malformations (CCMs) are severe vascular anomalies characterized by abnormal cerebrovascular development, leading to profound structural and functional deficits in the brain. However, the exact underlying mechanisms driving CCM pathogenesis remain incompletely understood. In this study, we identify a zebrafish *arap3* mutant that exhibits localized vascular dilation, cerebral hemorrhage, impaired vascular network formation and reduced mural cell coverage – phenotypes that collectively and strikingly recapitulate the key pathological features of clinical CCM. We demonstrate that *arap3* is highly enriched in cerebrovascular endothelial cells, and its deficiency severely impairs endothelial cell polarity and overall vascular homeostasis. Mechanistically, *arap3* contributes to CCM pathogenesis through the dysregulation of RhoA GTPase activity. Furthermore, we provide compelling evidence physically and functionally linking Arap3 to the CCM signaling complex by demonstrating that Arap3 interacts with Krit1 (CCM1). Our findings establish a crucial framework for understanding the etiology of CCM and present a highly trackable *in vivo* model for the future discovery of targeted therapeutics.

## Teaser

**Summary:** We identify *arap3* as a novel regulator of cerebrovascular development and establish a new zebrafish model of cerebral cavernous malformation.

## INTRODUCTION

Cerebral cavernous malformations (CCMs) are devastating vascular anomalies within the central nervous system, anatomically characterized by clusters of enlarged, thin-walled and hyperpermeable capillary channels that are frequently associated with deficient mural cell coverage and disrupted neurovascular unit organization (Baranoski et al., 2016; Snellings et al., 2021). These fragile lesions affect approximately 0.1-0.5% of the population and predispose individuals to severe clinical manifestations, including intracerebral hemorrhage, stroke, epilepsy and progressive focal neurological deficits, thereby representing a significant global health burden (Akers et al., 2009). Over the past 2 decades, seminal genetic studies have established that CCMs primarily arise from loss-of-function mutations in three core genes: *KRIT1* (CCM1), *CCM2* and *PDCD10* (CCM3) (Denier et al., 2006). The functional collapse of this CCM protein complex compromises endothelial cell-cell junctional integrity, precipitating profound barrier dysfunction (Hogan et al., 2008; Lampugnani et al., 2010; Liu et al., 2011a). This pathological remodeling is largely driven by the aberrant hyperactivation of downstream signaling cascades – most notably the RhoA/ROCK axis (Stockton et al., 2010; Faurobert et al., 2013; Liu et al., 2013; Shenkar et al., 2017), alongside dysregulated PI3K/AKT, Notch, WNT and TGFβ pathways (Maddaluno et al., 2013; Fisher et al., 2015a; DiStefano and Glading, 2020; Qi et al., 2023).

To decipher these intricate mechanisms, researchers have traditionally relied on genetically engineered murine models (Boulday et al., 2009; Chan et al., 2011; Phillips et al., 2022). While these mammals have provided invaluable insights, they harbor distinct limitations. For instance, homozygous deletion of CCM genes in mice typically results in early embryonic lethality due to catastrophic cardiovascular defects, mandating the use of complex, inducible systems to model postnatal lesions (Boulday et al., 2009; Chan et al., 2011). Moreover, the inherent opacity of the mammalian cranium and the protracted timeline of lesion genesis severely impede the real-time, high spatiotemporal resolution imaging of the precipitating cellular events (Abou-Fadel and Zhang, 2020; Phillips et al., 2022). These constraints underscore an urgent need for alternative vertebrate models that permit dynamic, high-throughput interrogation of cerebrovascular pathologies.

The zebrafish (*Danio rerio*) represents a powerful complementary model system for vascular disease research, owing to its external embryonic development, inherent optical transparency and highly conserved cardiovascular physiology (Hove, 2004; McKinney and Weinstein, 2008; Saleem and Kannan, 2018). Leveraging an N-ethyl-N-nitrosourea (ENU)-based forward genetic screen (Driever et al., 1996), we identified a zebrafish mutant exhibiting highly specific cerebrovascular defects and spontaneous intracerebral hemorrhage that closely recapitulate the pathological hallmarks of human CCM. Positional cloning identified the causative mutation in *arap3*, which encodes Arf-GAP with Rho-GAP domain, ankyrin repeat and PH domain 3 (Arap3).

As a dual GTPase-activating protein and downstream effector of phosphoinositide 3-kinase (PI3K) (Craig et al., 2010; Bao et al., 2016), Arap3 has emerged as a key regulator of vascular homeostasis (Krugmann et al., 2002). Previous studies have demonstrated that genetic deletion of *arap3* or impairment of its PI3K-dependent activation causes severe angiogenic defects during embryogenesis, supporting an endothelial cell-autonomous role in vascular morphogenesis (Gambardella et al., 2010). Arap3 has also been implicated in lymphatic vascular development and neutrophil adhesion and migration (Kartopawiro et al., 2014; McCormick et al., 2019). Despite these established roles in general vascular biology, the function of Arap3 in cerebrovascular development and its potential involvement in CCM pathogenesis remain entirely unexplored.

In this study, we characterize the zebrafish *arap3* mutant as a novel *in vivo* model of CCM that faithfully recapitulates key pathological features of the human disease, including focal cerebrovascular dilation, spontaneous hemorrhage, impaired vascular network formation and reduced mural cell coverage. We demonstrate that *arap3* is highly enriched in cerebrovascular endothelial cells and acts cell-autonomously to maintain endothelial polarity and vascular homeostasis. Mechanistically, we show that Arap3 physically interacts with the core CCM protein Krit1 (CCM1) and functions as a crucial negative regulator of RhoA GTPase activity.

## RESULTS

### The *vc29* mutant exhibits cerebral hemorrhage and developmental vascular defects reminiscent of CCM

Although extensive research has been conducted on the regulation of vascular development currently, the mechanisms specifically governing cerebrovascular development remain incompletely elucidated. To identify previously unreported factors essential for brain vascular development, we performed an ENU forward genetic screen and identified the *vc29* mutant, which exhibited cerebral hemorrhage at 48 h post fertilization (hpf) (Fig. 1A). To characterize the dynamics of this hemorrhagic event, we traced cellular behavior in the *vc29* mutant under the *Tg(kdrl:GFP;gata1:dsRed)* transgenic background, in which endothelial cells were labeled with GFP and erythrocytes with DsRed. At 36 hpf, we observed localized vascular dilatation accompanied by blood cell accumulation (Fig. 1B,C and Fig. S1A,B). By 48 hpf, these vessels ruptured, lost their original morphology and exhibited extensive blood cell leakage (Fig. 1B,D). Notably, trunk vascular development remained completely unaffected in *vc29* mutants (Fig. 1F), indicating a brain-specific vascular phenotype.

**Fig. 1.**
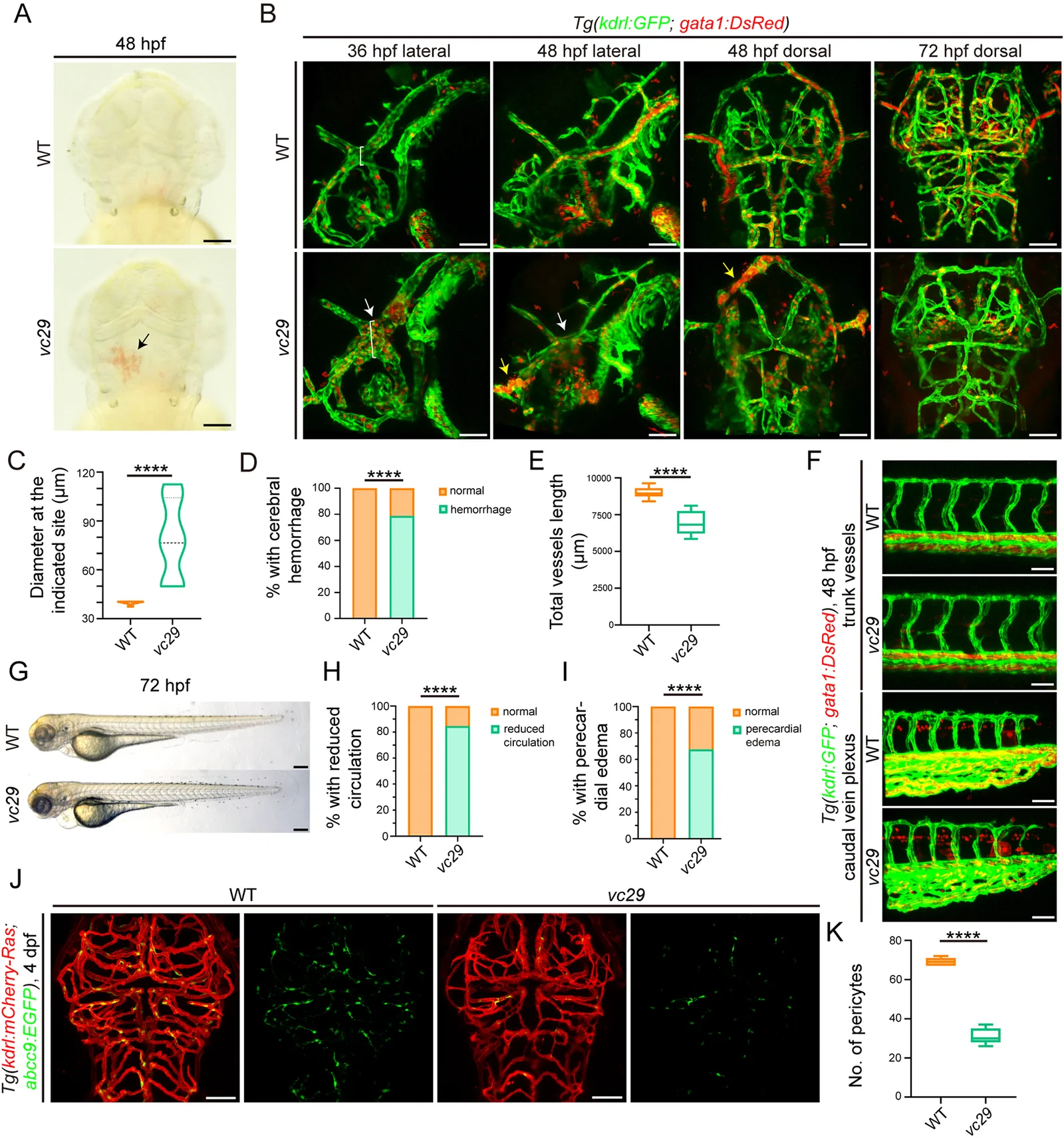
**The *vc29* mutant exhibits cerebral hemorrhage and developmental vascular defects reminiscent of CCM.** (A) Bright-field images of representative heads of wild-type and *vc29* mutant embryos at 48 hpf. The black arrow indicates the hemorrhagic site. (B) Lateral and dorsal views of the cerebral vasculature in wild-type and *vc29* mutant embryos of the *Tg(kdrl:EGFP; gata1:DsRed)* line at 36, 48 and 72 hpf. The white arrows indicate the progression from vessel dilation to rupture and hemorrhage at the same vascular site. The yellow arrows indicate erythrocyte accumulation caused by a vascular malformation at a different site at 72 hpf. White brackets indicate the vessel segments used for diameter quantification in C. (C) Violin plot showing the vessel diameter at the indicated sites in wild type (*n*=10) and *vc29* mutants (*n*=10) in B. (D) Incidence of cerebral hemorrhage in wild type (*n*=30) and *vc29* mutants (*n*=33) at 48 hpf. (E) Box plot showing the total cerebral vessel length in wild type (*n*=7) and *vc29* mutants (*n*=11) at 72 hpf. The center line indicates the median, the box indicates the interquartile range and the whiskers indicate the minimum and maximum values. (F) Confocal images of trunk vessels and the caudal vein plexus in wild-type and *vc29* mutant embryos of the *Tg(kdrl:EGFP; gata1:DsRed)* line at 48 hpf. (G) Bright-field images of representative whole wild-type and *vc29* mutant embryos at 72 hpf. Scale bar: 200 μm. (H) Incidence of reduced circulation in wild type (*n*=60) and vc29 mutants (*n*=59) at 5 dpf. (I) Incidence of pericardial edema in wild type (*n*=40) and *vc29* mutants (*n*=40) at 5 dpf. (J) Confocal images showing the cerebral vasculature and pericytes in the *Tg(kdrl:mCherry-Ras; abcc9:EGFP)* line at 4 dpf. (K) Box plot showing the number of pericyte in wild type (*n*=7) and vc29 mutants (*n*=11) at 4 dpf. The center line indicates the median, the box indicates the interquartile range and the whiskers indicate the minimum and maximum values. ****P<0.0001 (two-tailed unpaired *t*-test in C,E,K; chi-square test in D,H,I). Scale bars: 50 μm. dpf, days post-fertilization; hpf, hours post-fertilization; WT, wild type.

By 72 hpf, *vc29* mutants failed to establish a functional, intact cerebrovascular network (Fig. 1B,E), and concurrently displayed impaired systemic circulation and pericardial edema at 5 dpf (Fig. 1G-I). Notably, we found that the brain vascular dilation and hemorrhage were more likely to occur at branch points, and the location and severity of hemorrhage and the degree of vascular integrity varied considerably among embryos, following an approximately normal distribution (Fig. S1C,D). These cerebrovascular defects persisted throughout development, and the mutant embryos did not survive beyond 15 days post-fertilization (Fig. S1E-G). In addition, analysis using the transgenic line *Tg(abcc9:EGFP)* revealed a marked reduction in pericytes in the *vc29* mutant (Fig. 1J,K). Long-term time-lapse imaging indicated that the reduced pericyte coverage is likely attributable to impaired pericyte recruitment secondary to vascular abnormalities, rather than to a primary defect in pericyte development (Fig. S2A,B). Moreover, the expression of the blood-brain barrier marker *glut1b* was significantly reduced (Fig. S2C,D), and the junctional organization of Claudin 5 (Cldn5) was markedly disrupted (Fig. S2E), suggesting impaired blood-brain barrier maturation and integrity. Together, these phenotypes, including focal vascular dilation, cerebral hemorrhage, reduced vascular wall cell coverage and impaired blood-brain barrier integrity, are highly reminiscent of the pathological features of CCM. In summary, we identified a mutant with vascular developmental defects that closely resemble the clinical features of CCM.

### The CCM-like phenotype is caused by *arap3* deficiency

To identify the causative locus for this CCM-like phenotype, we crossed the *vc29* heterozygous with the polymorphic line SJD-C and performed fine-mapping analysis. The *vc29* mutant allele was mapped to chromosome 14, within the interval flanked by the simple sequence length polymorphism (SSLP) markers z7673 and z9057 (Fig. 2A). Subsequent sequencing revealed a T-to-A transversion at the splice junction of exon 8 in the *arap3* gene (Fig. 2B), causing complete skipping of exon 8, a frameshift and premature translational termination of Arap3 protein. The truncated mutant protein is predicted to lack the majority of its functional domains, including the crucial Rho-GAP domain (Fig. 2D).

**Fig. 2.**
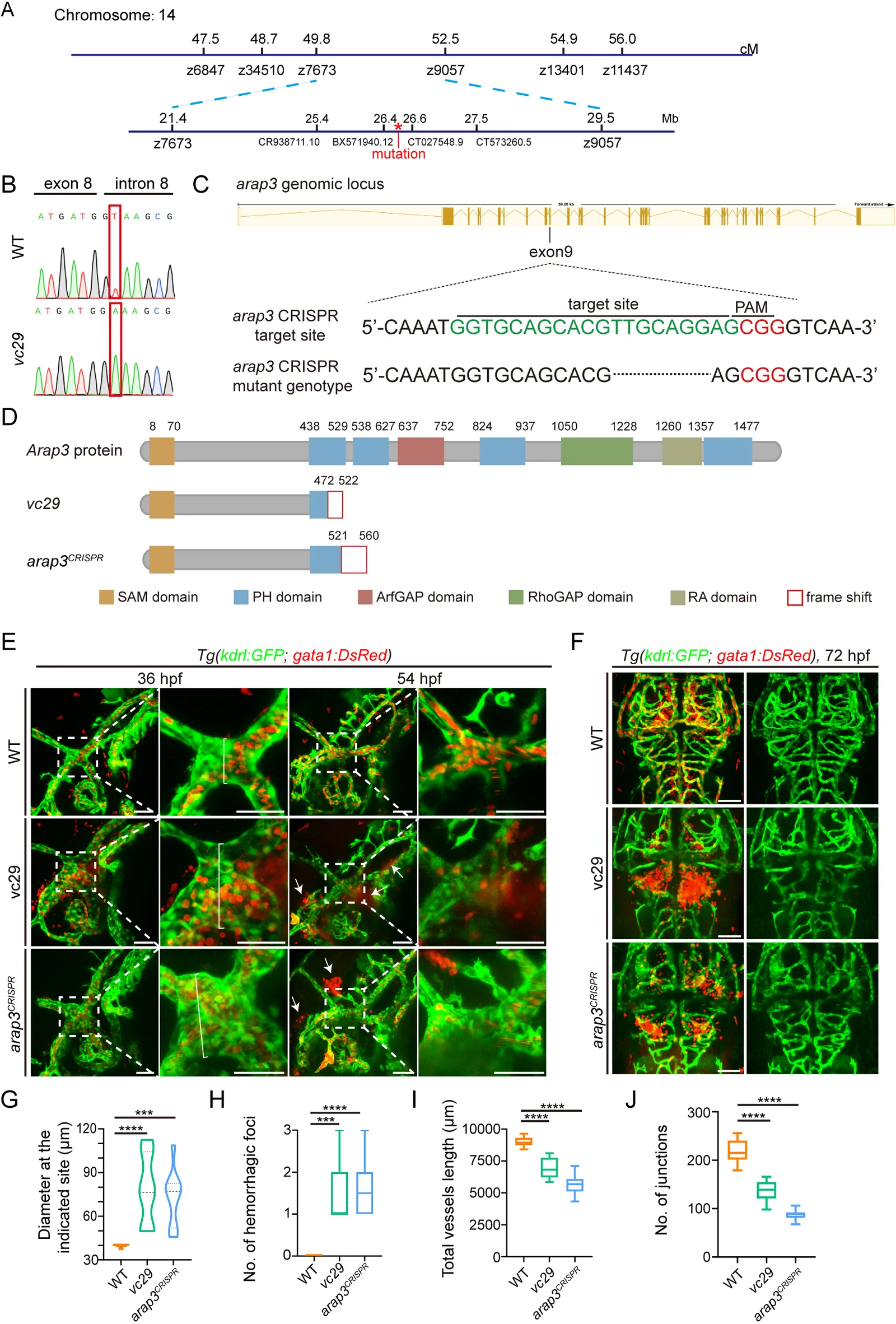
**The CCM-like phenotype is caused by *arap3* deficiency.** (A) Genetic map of the candidate region on chromosome 14 between SSLP markers z7673 and z9057, containing four open reading frames, with the causative mutation (red asterisk) mapped to the *arap3* gene. (B) Genomic sequencing results of *arap3* gene in wild type and *vc29* mutant. The T-A point mutation is outlined in red. (C) Schematic diagram of the *arap3* CRISPR/Cas9 target site (green) and the PAM sequence (red). The dotted line indicates the loss of 7 bases in the *arap3^CRISPR^* mutant. (D) Schematic diagram of the Arap3, *vc29* mutant and *arap3^CRISPR^* mutant proteins. (E) Images showing lateral sections of cerebral vascular of wild type, and *vc29* and *arap3^CRISPR^* mutants under the *Tg(kdrl:GFP;gata1:DsRed)* transgenic line at 36 hpf and 54 hpf. The white arrows indicate the hemorrhage point. Areas shown at higher magnification are outlined. White brackets indicate the vessel segments used for diameter quantification in G. (F) Images showing dorsal sections of cerebral vascular of wild type, and *vc29* and *arap3^CRISPR^* mutants under the *Tg(kdrl:GFP;gata1:DsRed)* transgenic line at 72 hpf. (G) Violin plots showing vessel diameter at the indicated sites of wild type (*n*=10), *vc29* mutant (*n*=10) and *arap3^CRISPR^* mutant (*n*=10) at the indicated site in E. (H) Quantification of the number of hemorrhagic foci of wild type (*n*=10), and *vc29* (*n*=10) and *arap3^CRISPR^* (*n*=10) mutants at 52 hpf. (I) Quantification of total length of cerebral vessels in wild type (*n*=7), and *vc29* (*n*=11) and *arap3^CRISPR^* (*n*=10) mutants at 72 hpf. (J) Quantification of the number of branches of wild type (*n*=8), and *vc29* (*n*=11) and *arap3^CRISPR^* (*n*=11) mutants at 72 hpf. Data in H-J are presented as box plots. The center line indicates the median, the box indicates the interquartile range, and the whiskers indicate the minimum and maximum values. In G, data are presented as mean±s.d. Statistical significance was assessed using one-way ANOVA followed by Holm–Šidák's multiple-comparisons test: ****P<0.0001, ***P<0.001. Scale bars: 50 μm. dpf, days post-fertilization; hpf, hours post-fertilization; PAM, protospacer adjacent motif; SSLP, simple sequence length polymorphism; WT, wild type.

To further confirm that loss of *arap3* drives the cerebrovascular developmental defects, we generated a new *arap3* mutant using CRISPR/Cas9 technology. We designed the gRNA target site within exon 9 and ultimately generated a mutant carrying a 7 bp deletion, which induced a frameshift and premature termination (designated *arap3^CRISPR^*; Fig. 2C,D). The *arap3^CRISPR^* mutants effectively phenocopied the *vc29* mutant, exhibiting localized vascular dilation at 36 hpf, vascular rupture and hemorrhage at 54 hpf, and failure to form an intact vascular network by 72 hpf (Fig. 2E-J).

Additionally, we constructed *Tg(hsp70l:arap3-P2A-EGFP)* transgenic line to enable heat shock-inducible overexpression of *arap3* (Fig. S3A,B). Heat shock induction at 32 hpf effectively rescued the mutant phenotype, with most mutants no longer displaying intracranial hemorrhage at 48 hpf (Fig. S3C,F) and subsequently developing a relatively normal cerebrovasculature (Fig. S3D,G-I). Collectively, these results confirm that the CCM-like phenotype is caused by *arap3* deficiency, and establish an essential previously unrecognized role for *arap3* in brain vascular development.

### *arap3* regulates cerebral vascular development in a cell-autonomous manner

To investigate the spatial expression pattern of *arap3* during development, we performed whole-mount *in situ* hybridization. We found that, from 24 hpf to 48 hpf, *arap3* was predominantly expressed in the early formed major blood vessels of the brain and trunk, with weaker expression detected in the neural crest (Fig. 3A). To further delineate the cell types in which *arap3* functions, we performed fluorescence *in situ* hybridization combined with antibody staining for endothelial markers. Confocal co-localization analysis confirmed that *arap3* is highly enriched within *kdrl*+ cerebrovascular endothelial cells (Fig. 3B). Independent analysis of a published zebrafish single-cell RNA-sequencing dataset (Farnsworth et al., 2020) further supported these findings, demonstrating that *arap3* is enriched in endothelial cells and exhibits an expression pattern highly consistent with canonical endothelial markers, including *kdrl*, *fli1a*, *flt1*, *pecam1a* and *tek* (Fig. 3C,D). Although *arap3* expression was also detected in a subset of neurons (Fig. 3D and Fig. S4A), no significant neuronal developmental defects were observed in *arap3* mutants (Fig. S4B).

**Fig. 3.**
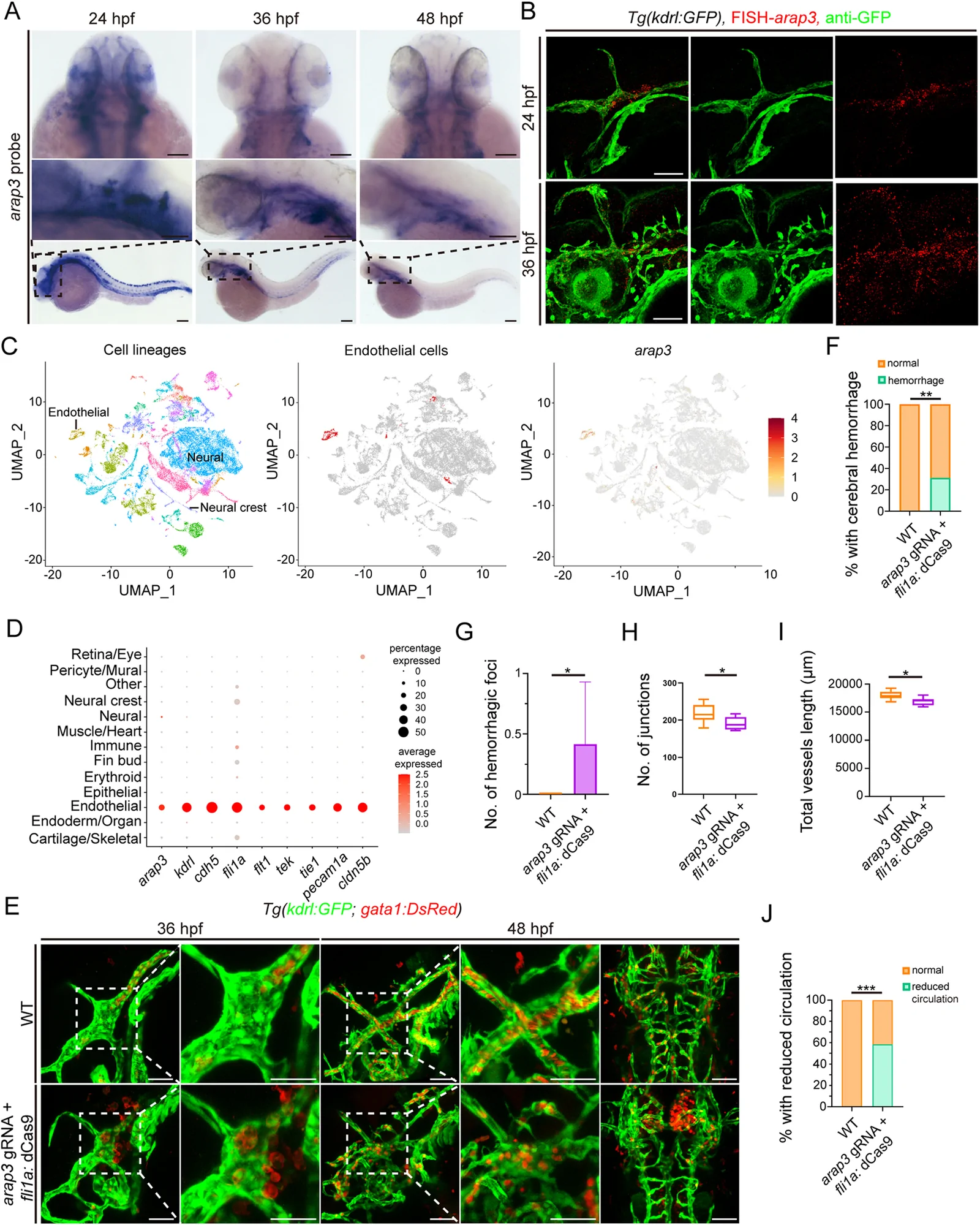
***arap3* regulates cerebral vascular development in a cell-autonomous manner.** (A) Whole-mount *in situ* hybridization results showing the expression of *arap3* at 24 hpf, 36 hpf and 48 hpf. Areas shown at higher magnification are outlined. Scale bars: 50 μm. (B) Fluorescent *in situ* hybridization results showing the expression of *arap3* at 24 hpf and 36 hpf. (C) UMAP embedding of all cells. Left, cells colored by lineage; middle, endothelial cells highlighted in red; right, *arap3* expression (log-normalized; color scale capped at the 99th percentile). *arap3^+^* cells localize to the endothelial territory. (D) Dot plot of *arap3* and endothelial markers across lineages. Dot size indicates the percentage of cells expressing the gene; color indicates average expression. *arap3* is expressed in 26.2% of endothelial cells versus <4.4% in every other lineage (∼10-fold enrichment), mirroring the eight canonical endothelial markers. (E) Confocal images showing the cerebral vasculature of wild-type embryos and embryos injected with *arap3* gRNA with *fli1a:dcas9* under the *Tg(kdrl:GFP;gata1:DsRed)* transgenic line at 36 hpf and 48 hpf. (F) Incidence of cerebral hemorrhage in wild type (*n*=30) and embryos injected with *arap3* gRNA with *fli1a:dcas9* (*n*=16) at 48 hpf. (G) Quantification of the number of hemorrhagic foci of wild type (*n*=10) and *arap3* gRNA with *fli1a:dcas9* (*n*=16) at 48 hpf. (H) Quantification of the number of branches of wild type (*n*=10) and *arap3* gRNA with *fli1a:dcas9* (*n*=16) at 72 hpf. (I) Quantification of total length of cerebral vessels in wild type (*n*=10) and *arap3* gRNA with *fli1a:dcas9* (*n*=16) at 72 hpf. (J) Incidence of reduced circulation in wild type (*n*=10) and *arap3* gRNA with *fli1a:dcas9* (*n*=16) at 5 dpf. Data in G are presented as mean±s.d, In H and I, the center line indicates the median, the box indicates the interquartile range, and the whiskers indicate the minimum and maximum values. *P<0.05, **P<0.01, ***P<0.001 (two-tailed unpaired *t*-test in G-I; the chi-square test in F and J). Scale bars: 50 μm.

Given its enriched expression in vascular endothelial cells, we next tested whether *arap3* acts in a cell-autonomous manner in endothelial cells to regulate cerebrovascular development. We employed a CRISPR-dCas9-KRAB system (Chang et al., 2013) to perform endothelial-specific *arap3* knockdown, driven by the *fli1a* promoter. Endothelial-specific knockdown of *arap3* was sufficient to recapitulate the cerebral hemorrhagic phenotype of *arap3^CRISPR^* mutants (Fig. 3E-J). Conversely, endothelial-specific re-expression of *arap3* using the *Tg(fli1a:Gal4FF;UAS:arap3)* transgenic line partially rescued the cerebrovascular defects in *arap3* mutants (Fig. S3E-I). These results indicate that the CCM-like phenotype of *arap3^CRISPR^* mutants is primarily attributable to the loss of *arap3* in endothelial cells, supporting a cell-autonomous role of *arap3* in brain vascular development.

### *arap3* is required for endothelial cell polarity and vascular homeostasis

To investigate the developmental basis of the cerebral hemorrhage phenotype in *arap3^CRISPR^* mutants, we performed real-time time-lapse imaging of the cerebrovasculature starting at 34 hpf. We found that angiogenesis from major hemorrhagic branch points, such as the middle cerebral vein (MCeV), was severely abnormal, with nascent sprouts regressing during development (Fig. 4A,B, white arrows). Quantification at 38 hpf further demonstrated a significant reduction in the number of angiogenic sprouts from the PHBC and PMBC in *arap3^CRISPR^* mutants compared with controls (Fig. 4C). In contrast, angiogenic sprouting from the dorsal aorta (DA) was unaffected (Fig. S5A,B), consistent with the absence of overt trunk vascular defects observed at 48 hpf (Fig. 1F). Hemorrhage consistently occurred subsequent to these vascular morphological abnormalities (Fig. 4B, yellow arrowhead), demonstrating that hemorrhage occurred secondary to vascular defects.

**Fig. 4.**
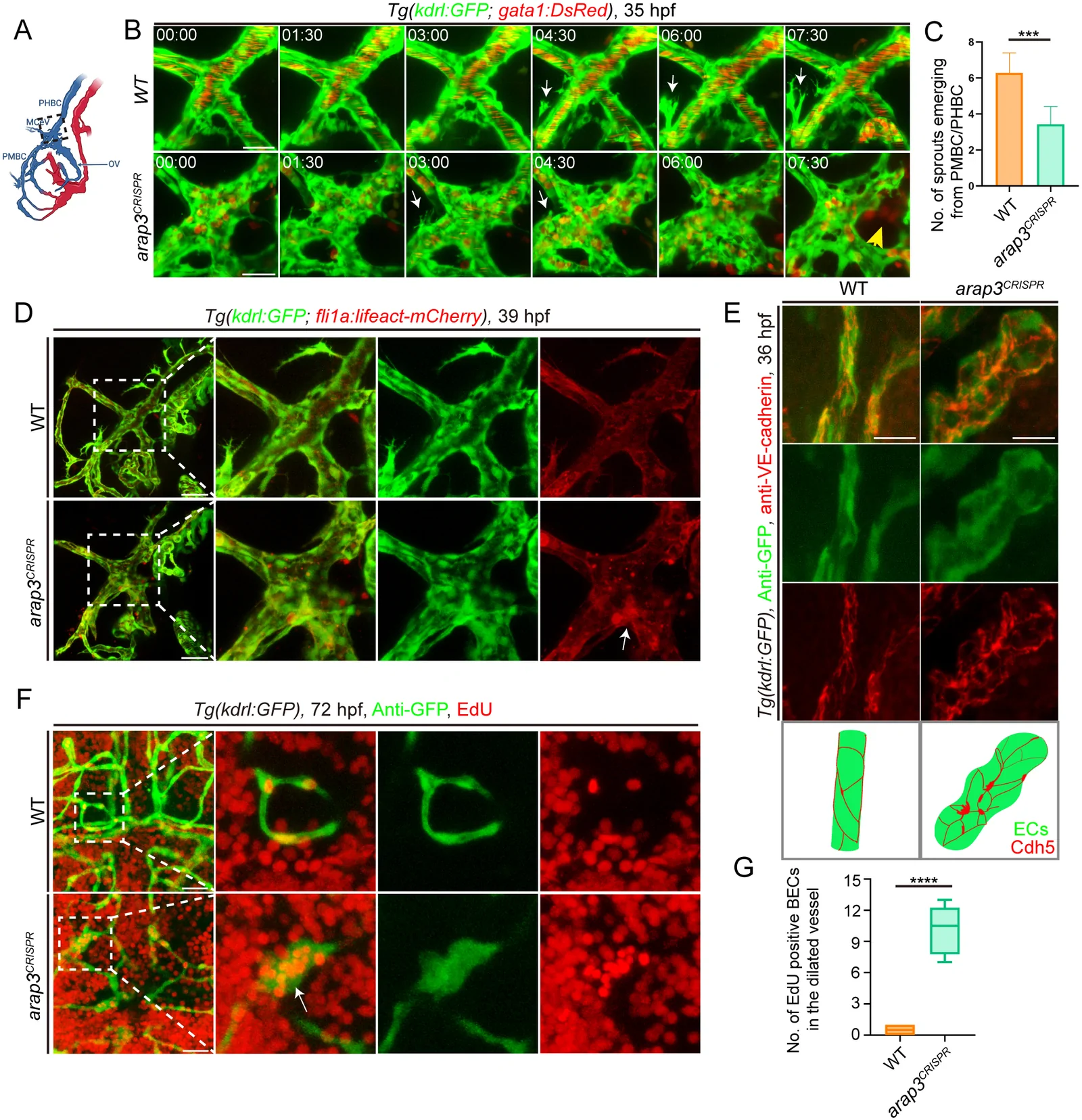
**Loss of *arap3* disrupts endothelial polarity and vascular homeostasis.** (A) Schematic diagram of the vasculature in a 36 hpf zebrafish embryo. The region shown in B is outlined. (B) Time-lapse imaging of the cerebral vasculature in wild-type and *arap3^CRISPR^* embryos. The white arrows indicate the angiogenic sprouting site. The yellow arrow indicates the hemorrhagic foci. (C) Quantification of the number of sprouts emerge from PHBC/PMBC of wild type (*n*=6) and *arap3^CRISPR^* mutant (*n*=6). Data are mean±s.d. (D) Confocal images showing endothelial F-actin distribution in wild-type and *arap3^CRISPR^* embryos of the *Tg(fli1a:Lifeact-mCherry)* line at 39 hpf. Areas shown at higher magnification are outlined. The white arrow indicates disorganized F-actin. (E) Confocal images and a schematic illustration showing the distribution of VE-cadherin at endothelial cell–cell junctions in wild-type and *arap3^CRISPR^* embryos. (F) EdU assay of embryos injected at 32 hpf and fixed at 72 hpf. Areas shown at higher magnification are outlined. The white arrow indicates EdU signal accumulation within the dilated vessel. (G) Box plot showing the number of EdU-positive endothelial cells in dilated vessels of wild type (*n*=6) and *arap3^CRISPR^* mutant (*n*=6) embryos. The center line indicates the median, the box indicates the interquartile range, and the whiskers indicate the minimum and maximum values. ****P<0.0001, ***P<0.001 (two-tailed unpaired *t*-test). Scale bars: 50 μm. BECs, blood vascular endothelial cells; EdU, 5-ethynyl-2′-deoxyuridine; hpf, hours post-fertilization; MceV, middle cerebral vein; OV, optic vein; PHBC, primordial hindbrain channel; PMBC, primordial midbrain channel; WT, wild type.

To further characterize the cellular basis of these defects, we analyzed endothelial F-actin organization using *Tg(fli1a:Lifeact-mCherry)*, and found that F-actin was uniformly distributed in wild-type embryos but became disorganized in the *arap3^CRISPR^* mutants (Fig. 4D). We next examined the junctional organization of vascular endothelial cadherin (VE-cadherin, encoded by *cdh5*). In wild-type embryos, VE-cadherin localized to endothelial cell-cell junctions in a continuous, ring-like pattern, whereas in the dilated mutant vessels, it exhibited focal, patch-like accumulation with disrupted junctional integrity (Fig. 4E). Together, the disorganized F-actin and disrupted VE-cadherin junctions are consistent with impaired endothelial cell polarity during cerebrovascular angiogenesis (Phng et al., 2015).

Furthermore, 5-ethynyl-2′-deoxyuridine (EdU) labeling revealed an overall reduction in endothelial cell proliferation in *arap3^CRISPR^* mutants. Despite this decrease, EdU-positive endothelial cells were preferentially enriched within the dilated vessel segments (Fig. 4F,G). We also detected significantly increased apoptotic signals in the blood vascular endothelial cells (BECs) of *arap3^CRISPR^* mutants via terminal deoxynucleotidyl transferase dUTP nick end labeling (TUNEL) staining (Fig. S5C,D). Collectively, these findings indicate that endothelial homeostasis is profoundly disrupted in the *arap3^CRISPR^* mutants, as reflected by abnormal cytoskeletal organization, altered junctional distribution, reduced endothelial cell proliferation accompanied by focal accumulation of proliferating endothelial cells in dilated vessels and increased apoptosis.

### *arap3* maintains cerebrovascular stability by restraining RhoA hyperactivation

Given the established role of RhoA signaling in coordinating endothelial cytoskeletal organization, junctional integrity and vascular homeostasis (Amano et al., 2010; Phng et al., 2015; Bao et al., 2016), we next examined whether RhoA activity is altered in *arap3^CRISPR^* mutants. Using an active RhoA pull-down assay, we found that GTP-bound active RhoA was markedly increased in the *arap3^CRISPR^* mutant embryo lysates compared to wild type, whereas total RhoA levels showed no obvious change (Fig. 5A,B). This finding indicates that *arap3* deficiency specifically leads to abnormal RhoA hyperactivation, rather than altering its overall expression, strongly supporting the notion that *arap3* acts as a negative regulator of RhoA activity in the developing cerebrovascular system.

**Fig. 5.**
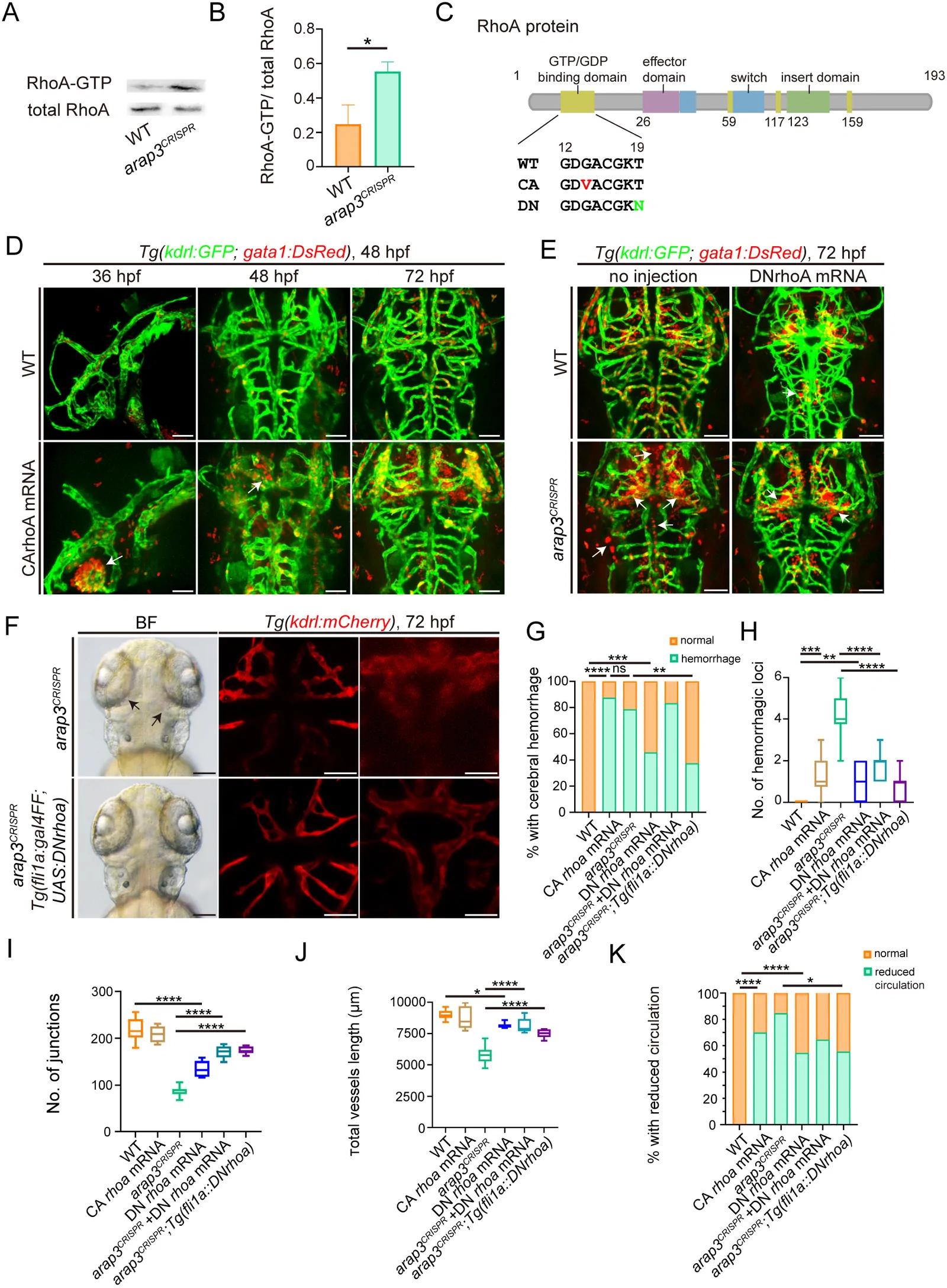
***arap3* maintains cerebrovascular stability by restraining RhoA hyperactivation.** (A) Representative western blot showing GTP-bound active RhoA and total RhoA in lysates from wild-type and *arap3^CRISPR^* embryos at 54 hpf. (B) Quantification of RhoA-GTP levels normalized to total RhoA (*n*=3). Data are mean±s.d. (C) Schematic representation of RhoA and its functional mutant variants. (D) Confocal images showing the cerebral vasculature in wild-type embryos and embryos injected with CArhoA mRNA in the *Tg(kdrl:EGFP; gata1:DsRed)* line at 36, 48 and 72 hpf. The white arrows indicate the hemorrhagic loci. (E) Confocal images showing the cerebral vasculature in wild-type embryos and *arap3^CRISPR^* embryos injected with DNrhoA mRNA at 72 hpf. The white arrows indicate the hemorrhagic loci. (F) Bright-field and *Tg(kdrl:mCherry)* fluorescence images of *arap3^CRISPR^* mutants and *arap3^CRISPR^* mutants with endothelial *arap3* rescue [*Tg(fli1a:gal4FF;UAS:arap3)*]*.* (G-K) Quantification across six experimental groups: wild-type, *CA-RhoA* mRNA-injected, *arap3^CRISPR^*, *DN-RhoA* mRNA-injected, *DN-RhoA* mRNA-injected *arap3^CRISPR^* and *arap3^CRISPR^*;*Tg(fli1a:gal4FF;UAS:arap3)* embryos. (G) Cerebral hemorrhage incidence at 48 hpf, [*n*=7 (WT), 8 (CA *rhoa*), 10 (*arap3^CRISPR^*), 8 (DN *rhoa*), 7 (*arap3^CRISPR^*+DN *rhoa*) and 6 (*arap3^CRISPR^*;*Tg(fli1a::DNrhoa)*)]. (H) Number of hemorrhagic foci at 72 hpf (*n*=15, 8, 10, 6, 7 and 6, as indicated previously). (I) Number of vascular junctions at 72 hpf (*n*=7, 8, 10, 6, 6 and 6, as indicated previously). (J) Total cerebral vessel length at 72 hpf (*n*=7, 8, 10, 6, 6 and 6, as indicated previously). (K) Reduced circulation incidence at 5 dpf (*n*=7, 8, 10, 6, 6 and 6, as indicated previously). In G and K, statistical significance was assessed using the chi-square test. In H-J, the center line indicates the median, the box indicates the interquartile range, and the whiskers indicate the minimum and maximum values; statistical significance was assessed using one-way ANOVA followed by Holm–Šidák's multiple-comparisons test. *P<0.05, **P<0.01, ***P<0.001, ****P<0.0001; ns, not significant. Scale bars: 50 μm. BF, bright field; dpf, days post-fertilization; hpf, hours post-fertilization; WT, wild type.

To further confirm the causal link between RhoA hyperactivation and the *arap3* mutant phenotype, we generated capped mRNAs encoding previously characterized constitutively active RhoA (CA-rhoa) and dominant-negative RhoA (DN-rhoa) based on published sequences (Fig. 5C) (Pillay et al., 2022) followed by microinjection into zebrafish embryos. Strikingly, microinjection of *CA-rhoa* mRNA into wild-type embryos faithfully phenocopied the *arap3* mutant defects, including focal vascular dilation at 36 hpf, cerebral hemorrhage by 48 hpf and defective cerebrovascular development at 72 hpf (Fig. 5D,G-K). Conversely, suppression of RhoA signaling via microinjection of *DN-rhoa* mRNA partially rescued the cerebrovascular defects in *arap3^CRISPR^* mutants, as reflected by fewer hemorrhagic foci, and improved cerebrovascular development (Fig. 5E,G-K). Pharmacological inhibition of the downstream RhoA effector ROCK with Rockout resulted in a near-complete rescue of cerebrovascular defects in the *arap3^CRISPR^* mutant (Fig. S6A-G), further supporting the conclusion that excessive RhoA/ROCK signaling is a major driver of the mutant phenotype. Consistent with these findings, endothelial-specific expression of DN-rhoa using the *Tg(fli1a:Gal4FF;UAS:DN-rhoa)* transgenic line also partially rescued the mutant phenotype (Fig. 5F-K), further supporting the conclusion that excessive endothelial RhoA activity contributes to the cerebrovascular defects caused by *arap3* deficiency. Together, these findings support the conclusion that excessive endothelial RhoA signaling contributes to the cerebrovascular defects caused by *arap3* deficiency. The striking phenotypic similarity to CCM and the convergence on RhoA dysregulation suggest that *arap3* may represent a previously unrecognized regulator linked to CCM-associated vascular instability.

### Arap3 physically and functionally interacts with Krit1 (CCM1)

The phenotypic similarity between *arap3^CRISPR^* mutants and CCM-associated vascular abnormalities, together with their shared association with dysregulated RhoA signaling (Liu et al., 2013; Shenkar et al., 2017; Snellings et al., 2021), led us to investigate whether *arap3* is functionally connected to canonical CCM pathways. As KRIT1 (CCM1) is a core component of the CCM complex and is essential for maintaining endothelial vascular stability, we used the endothelial-specific CRISPR-dCas9-KRAB system to knock down *krit1* in zebrafish endothelial cells. Endothelial-specific depletion of *krit1* resulted in a cerebral hemorrhagic phenotype at 48 hpf and impaired cerebrovascular development at 72 hpf (Fig. 6A-E), which closely resembled that induced by endothelial-specific *arap3* knockdown (Fig. 3E-J). Previous studies have reported that Arap3 binds PI(3,4)P_2_ and PI(3,4,5)P_3_ via its PH domains (Craig et al., 2010), and Krit1, as a core component of the CCM complex, is also localized to the endothelial plasma membrane (Liu et al., 2011b, 2013). Their shared subcellular localization suggested a potential functional association between the two proteins.

**Fig. 6.**
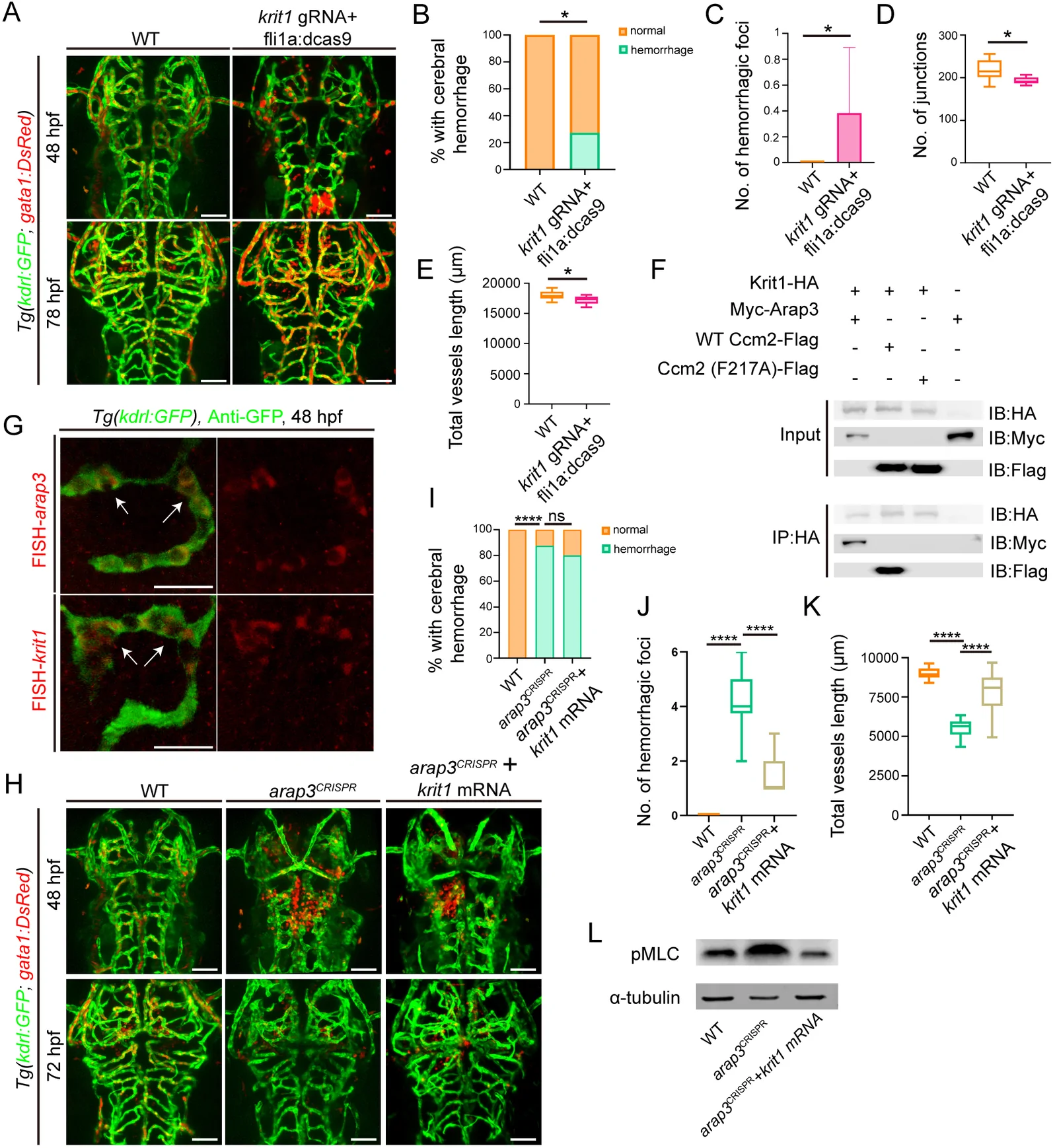
**Arap3 physically and functionally interacts with Krit1 (CCM1).** (A) Confocal images showing the cerebral vasculature of embryos injected with *arap3* gRNA or *krit1* gRNA together with *fli1a:dCas9-KRAB* in the *Tg(kdrl:EGFP; gata1:DsRed)* line at 48 hpf and 78 hpf. (B) Incidence of cerebral hemorrhage in wild-type embryos (*n*=30) and embryos injected with *krit1* gRNA together with *fli1a:dCas9-KRAB* (*n*=11) at 48 hpf. (C) Quantification of the number of hemorrhagic foci of wild type (n=30) and of embryos injected with *krit1* gRNA together with *fli1a:dCas9-KRAB* (*n*=11) at 72 hpf. (D) Quantification of the number of junctions of wild type (*n*=30) and embryos injected with *krit1* gRNA together with *fli1a:dCas9-KRAB* (*n*=11) at 72 hpf. (E) Box plot showing the total cerebral vessel length of wild type (*n*=30) and embryos injected with *krit1* gRNA together with *fli1a:dCas9-KRAB* (*n*=11) at 72 hpf. (F) Co-immunoprecipitation analysis of KRIT1-HA, Myc-ARAP3, wild-type CCM2-Flag and CCM2(F217A)-Flag. (G) Fluorescence *in situ* hybridization showing the expression of *arap3* and *krit1* in brain blood endothelial cells. (H) Confocal images showing the cerebral vasculature in wild-type embryos, *arap3^CRISPR^* embryos and *arap3^CRISPR^* embryos injected with *krit1* mRNA in the *Tg(kdrl:EGFP; gata1:DsRed)* line at 48 hpf and 72 hpf. (I) Incidence of cerebral hemorrhage in wild-type embryos (*n*=8), *arap3^CRISPR^* mutants (*n*=22) and *arap3^CRISPR^* embryos injected with *krit1* mRNA (*n*=31) at 48 hpf. (J) Box plot showing the number of hemorrhagic foci of wild-type embryos (*n*=8), *arap3^CRISPR^* mutants (*n*=22) and *arap3^CRISPR^* embryos injected with *krit1* mRNA (*n*=31) at 72 hpf. (K) Box plot showing the total cerebral vessel length in wild-type embryos (*n*=8), *arap3^CRISPR^* mutants (*n*=22) and *arap3^CRISPR^* embryos injected with *krit1* mRNA (*n*=31) at 72 hpf. (L) Western blot analysis of pMLC protein levels in wild-type embryos, *arap3^CRISPR^* mutants and *arap3^CRISPR^* embryos injected with *krit1* mRNA. In D,E,J,K, the center line indicates the median, the box indicates the interquartile range, and the whiskers indicate the minimum and maximum values. Statistical significance was assessed using the chi-square test (B,I) two-tailed unpaired Student's *t*-tests (C-E), one-way ANOVA followed by Holm–Šidák's multiple-comparisons test (J,K). *P<0.05, ****P<0.0001; ns, not significant. Scale bars: 50 μm. hpf, hours post-fertilization; WT, wild type.

To verify this interaction, we constructed *pcDNA3.1-Krit1-2HA* and *pcDNA3.1-Myc-Arap3* expression plasmids and co-transfected them into HEK293T cells. In parallel, *pcDNA3.1-CCM2-Flag* and the KRIT1-binding-defective mutant *pcDNA3.1-CCM2 (F217A)-Flag* (Fisher et al., 2015b) were used as positive and negative controls, respectively. Co-immunoprecipitation (co-IP) assays and western blot analysis showed that Arap3 interacted with Krit1, whereas CCM2-F217A failed to associate with Krit1 compared with wild-type CCM2, confirming the specificity of the interaction assay (Fig. 6F). To further determine whether *arap3* and *krit1* are expressed in the same cellular context, we performed fluorescence in situ hybridization analysis in zebrafish embryos. Similar to *arap3*, *krit1* expression was detected in cerebrovascular endothelial cells (Fig. 6G), supporting their potential functional relationship in endothelial cells. To determine whether Krit1 can functionally compensate for Arap3 deficiency, we *in vitro* transcribed *krit1* mRNA and injected it into *arap3^CRISPR^* mutant embryos at the one-cell stage. Time-lapse live imaging from 48 hpf to 72 hpf revealed that overexpression of *krit1* partially rescued the hemorrhage phenotype and significantly restored cerebrovascular development in *arap3^CRISPR^* mutants (Fig. 6H-K). We next examined pMLC levels as a readout of RhoA/ROCK activity. Consistent with the rescue of vascular defects, *krit1* overexpression reduced the elevated pMLC levels observed in *arap3^CRISPR^* mutants, suggesting that *krit1*-mediated rescue is associated with attenuation of aberrant RhoA/ROCK signaling (Fig. 6L). Collectively, these findings support a physical and functional association between Arap3 and the CCM signaling pathway, confirming that the *arap3^CRISPR^* mutant phenotype is a bona fide CCM-like pathology (Fig. S7).

## DISCUSSION

In this study, we highlight two major findings that advance our understanding of cerebrovascular development and disease. First, we successfully identified and characterized a zebrafish mutant that faithfully phenocopies human CCM *in vivo*. This model provides a unique platform for the real-time dynamic observation of cerebrovascular lesion progression, and offers an invaluable *in vivo* system for future high-throughput small-molecule drug screening to identify potential therapeutic targets. Second, we uncovered a previously unrecognized, brain-specific role for *arap3* in cerebrovascular development and homeostasis. While previous murine studies have demonstrated that *Arap3* knockout exacerbates inflammation-induced microvascular leakage and its downregulation is associated with hypotrichosis-lymphedema-telangiectasia (HLT)-like phenotypes, establishing its general requirement for embryonic vascular development (Kartopawiro et al., 2014), our study reveals that *arap3* mutation in zebrafish leads to severe localized cerebrovascular abnormalities and loss of homeostasis, while sparing the trunk vasculature. This work therefore defines a highly specific, brain-specific role for *arap3* in vascular development that has not been previously reported.

Animal models of CCM have provided important insights into the mechanisms underlying cerebrovascular instability. In zebrafish, Loss of *krit1* results in endothelial instability, vascular dilation and increased vascular permeability, with lesion formation influenced by hemodynamic forces (Roedel et al., 2019). Loss of *ccm2* has been shown to disrupt vascular integrity and cardiovascular development, whereas mosaic inactivation of *ccm2* generates cavernous vascular lesions resembling human CCM, characterized by focal vessel dilation and blood-filled vascular abnormalities (Mably et al., 2006; Li et al., 2021). However, unlike these CCM models, which exhibit severe systemic vascular defects leading to early lethality or lesion formation preferentially associated with low-flow regions, *arap3* mutants survive beyond early embryonic stages and display a predominantly cerebrovascular phenotype without obvious trunk vascular abnormalities. In contrast to global *Krit1* knockout mice, which exhibit severe systemic vascular defects and early embryonic lethality (Whitehead et al., 2004), *arap3* mutants survive beyond early embryonic stages, allowing the progression of cerebrovascular abnormalities to be examined during later developmental stages. Therefore, the *arap3* mutant provides a complementary model to investigate how dysregulated small GTPase signaling contributes to CCM-like vascular instability.

Previous studies have shown that KRIT1 regulates endothelial RhoA activity through multiple mechanisms, including its interaction with ICAP1, a regulator of integrin signaling. Loss of KRIT1 disrupts the KRIT1–ICAP1 complex, leading to aberrant integrin activation and increased RhoA/ROCK signaling, thereby compromising endothelial junction stability and vascular integrity (Faurobert et al., 2013; Liu et al., 2013). As ARAP3 directly functions as a RhoGAP toward RhoA, our findings suggest that ARAP3 may represent an additional layer of RhoA regulation within this network. The interaction between ARAP3 and KRIT1 raises the possibility that these proteins cooperate to fine-tune endothelial RhoA activity, although the precise molecular hierarchy among ARAP3, KRIT1 and other RhoA regulators remains to be determined.

Previous studies have demonstrated that dysregulated RhoA signaling plays a crucial role in vascular development and endothelial function. In particular, Pillay et al. (2022) showed that altered RhoA activity, through expression of constitutively active or dominant-negative RhoA variants, profoundly affects endothelial behavior and vascular morphogenesis in zebrafish. Consistent with these findings, our study demonstrates that increased RhoA activity is sufficient to induce cerebrovascular dilation and hemorrhage, as expression of CA-rhoa phenocopied key features of the *arap3* mutant phenotype. Conversely, inhibition of RhoA signaling through DN-rhoa expression or ROCK inhibition partially to strongly rescued vascular defects caused by *arap3* deficiency. Together, these results extend previous findings by identifying ARAP3, a RhoA GAP, as an upstream regulator required to maintain endothelial RhoA homeostasis during cerebrovascular development.

While the regulation of RhoA activity is a well-established and classic downstream pathway in CCM pathogenesis, our study identifies *arap3* as a previously unreported upstream regulator of this cascade, linking it directly to the canonical CCM complex via interaction with Krit1. In future studies, it will be crucial to investigate how *arap3* is dynamically recruited to specific subcellular membrane microdomains during cerebrovascular development. For example, determining whether this membrane targeting is strictly dependent on the interaction between its PH domains and the PI3K signaling pathway will provide deeper mechanistic insights into its spatiotemporal regulation during vascular development.

Additionally, our study highlights a direct physical interaction between Arap3 and Krit1 (CCM1), bridging *arap3* to the classical CCM complex. However, the current co-immunoprecipitation (Co-IP) evidence was obtained using an *in vitro* overexpression system in HEK293T cells. To further solidify the clinical and biological relevance of this interaction, future studies must prioritize demonstrating this complex formation under physiological conditions, utilizing endogenous Co-IP assays in endothelial cells and *in vivo* co-localization imaging in our zebrafish model.

### Study limitations

There are several important limitations to this study that should be noted. First, while our zebrafish model faithfully recapitulates the early developmental vascular defects of CCM, we have not characterized the long-term progression of lesions in juvenile and adult fish, which will be crucial to validate its utility for modeling chronic CCM disease. Second, the physical interaction between Arap3 and Krit1 was demonstrated in an overexpression system, and future work will be required to confirm this endogenous interaction in endothelial cells and define the precise protein domains mediating this binding. Third, while we show that RhoA hyperactivation is the key downstream effector of *arap3* deficiency, we have not fully excluded the contribution of Arf GTPase dysregulation, given that Arap3 is a dual GAP protein; future studies will explore the role of Arf signaling in this CCM model. Finally, while our model shows promise for drug screening, we have not yet performed a proof-of-concept small-molecule screen to validate its scalability and utility for therapeutic discovery.

In conclusion, we demonstrate that *arap3* is enriched in cerebrovascular endothelial cells and is essential for maintaining endothelial polarity and vascular homeostasis. Our findings support a physical and functional association between Arap3 and the CCM signaling complex through its interaction with Krit1 (CCM1), and further indicate that *arap3* contributes to CCM pathogenesis through dysregulation of RhoA activity. The *arap3* mutant established in this study represents a powerful *in vivo* model for CCM research. Exploiting the inherent advantages of the zebrafish system – namely its optical transparency and rapid *ex utero* development – this CCM-like model not only enables unprecedented live imaging of vascular lesion pathogenesis but also serves as an ideal, cost-effective platform for large-scale *in vivo* pharmacological screens, paving the way for the discovery of potential therapeutic interventions for CCM.

## MATERIALS AND METHODS

### Ethical approval

The zebrafish facility and all experimental procedures were approved by the Institutional Review Board of Southwest University (Chongqing, China). Zebrafish were maintained in compliance with the Guidelines of Experimental Animal Welfare from Ministry of Science and Technology of People's Republic of China (2006). All animal experiments were conducted in accordance with protocols approved by the Institutional Animal Care and Use Committee of Southwest University (IACUC, 2007). The IACUC number is: IACUC-20230309-07.

### Zebrafish strains

The following zebrafish (*Danio rerio*) lines were used in this study: *arap3^CRISPR^* mutant, *Tg(kdrl:GFP)^S843^*, *Tg(gata1:DsRed)^sd2^*, *Tg(kdrl:CFP-nitroreductase(NTR))^cq62^*, *Tg(fli1a:Lifeact-mCherry)*, *Tg(kdrl:mCherry)^cq15^*, *Tg(huc:EGFP)*, *Tg(abcc9:EGFP)* and *Tg(hsp70l:arap3-P2A-EGFP)*. Zebrafish embryos and larvae were raised in egg water at 28.5°C, and 0.003% 1-phenyl-2-thiourea (PTU, Sigma-Aldrich, USA) was added to the egg water to inhibit pigment formation. Embryos were staged by hours post fertilization (hpf) and days post fertilization (dpf) according to standard morphological criteria.

### ENU mutagenesis and positional cloning

ENU (Sigma) mutagenesis was performed following established protocols (Solnica-Krezel et al., 1994). Adult male zebrafish were exposed to 3.5 mM ENU for 1 h once per week over a period of 6 weeks. After a 2-week recovery period following the final ENU treatment, mutagenized males were outcrossed with wild-type females to generate F1 families. F1 heterozygous carriers from the *cq* line were subsequently crossed with the polymorphic SJD strain to obtain F2 progeny. F2 heterozygotes were identified and intercrossed to produce offspring that served as the mapping population. Chromosomal localization of the mutation was carried out using a standard panel of simple sequence length polymorphism (SSLP) markers. Total RNA was extracted from whole zebrafish embryos using the RNeasy kit (Qiagen) according to the manufacturer's instructions. First-strand cDNA was synthesized with the SuperScript III Reverse Transcriptase kit (Invitrogen) following the supplier's protocol.

### CRISPR/Cas9 system-mediated mutagenesis

CRISPR/Cas9-mediated genome editing was conducted according to previously published methods (Chang et al., 2013). Cas9 mRNA (300 pg) and *arap3* gRNA (50 pg) were co-injected into zebrafish embryos at the one-cell stage. Genomic DNA encompassing the gRNA target site was subsequently amplified using gene-specific primers (forward, CTAGCCTGCACTTGGACCGATG; reverse, CACCTGCTCACTCTTGTACAAC) and sequenced to confirm mutagenesis. Successfully validated embryos were raised to adulthood (F0 generation). Founder fish were identified by screening F0 adults for germline transmission of insertions or deletions (indels) in the *arap3* locus. For genotyping, genomic DNA from CRISPR-derived embryos was amplified using the same primer set, and PCR products were analyzed by high-resolution gel electrophoresis followed by DNA sequencing.

### Whole-mount *in situ* hybridization, fluorescence *in situ* hybridization and antibody staining

Whole-mount *in situ* hybridization was carried out following established protocols (Thisse and Thisse, 2014). Larvae were fixed overnight at 4°C in 4% paraformaldehyde (PFA) prepared in PBS. Antisense RNA probes for *arap3* and *krit1* were used for whole-mount in situ hybridization analysis. Primers used for amplifying probe were: *arap3*, 5′-CATGTTAGACCCACAGCAGT-3′ and 5′-CATGGTGGAAAGTTCCTG-3′.

Fluorescent *in situ* hybridization alone and combined antibody staining were performed as previously described (He et al., 2014) using the *arap3* probe. Antisense probe labeled with digoxigenin or fluorescein, and anti-digoxigenin-POD (1:1000, Roche, 11207733910), anti-fluorescein-POD (1:1000, Roche), anti-GFP antibodies (1:1000, Abcam, ab6658) and anti-zebrafish-VE-cadherin antibodies (1:200, a gift from Markus Affolter, Biozentrum, University of Basel, Switzerland) and tyramide signal amplification and fluorescence detection system (TSA, PerkinElmer) were used to detect and amplify RNA *in situ* hybridization signals. Whole-mount antibody staining was performed using anti-GFP (1:1000, Abcam, ab6658), anti-zebrafish- VE-cadherin antibodies (1:200, a gift from Markus Affolter), anti-mCherry (1:1000, Abcam, ab125096), anti-pMLC (1:1000, Cell Signaling, 3674) and anti-Claudin5b (1:1000, Invitrogen, 18-7364) antibodies. The secondary antibodies used in the study were donkey anti-goat IgG Alexa fluor 488-conjugated (1:1000, Invitrogen, A11055) and goat anti-rabbit IgG Alexa fluor 405-conjugated (1:1000, Invitrogen, A31556).

### Endothelial-specific CRISPR/dCas9-KRAB knockdown

Endothelial-specific knockdown was achieved by co-injecting fli1a:dCas9-KRAB and sgRNA into zebrafish embryos at the one-cell stage. The target sequence of *arap3* gRNA 5′-GGTCCGTGCTCAGATGCAGAGG-3′ (PAM site underlined) was located in exon 2. The target sequence of *krit1* gRNA 5′-GATGGGAAACCAAGAGCTAGAGG-3′ (PAM site underlined) was located in exon 2. gRNAs were synthesized using the T7/Sp6 Transcription Kit (Ambion) according to the manufacturer's instructions (Chang et al., 2013).

### Analysis of published single-cell RNA-sequencing data

We analysed the published zebrafish whole-embryo single-cell transcriptome atlas (Farnsworth et al., 2020; NCBI BioProject PRJNA564810), comprising 44,020 cells from zebrafish at 1, 2 and 5 days post-fertilization. Data were retrieved from the UCSC Cell Browser (dataset: zebrafish-dev). The original UMAP coordinates and cluster annotations were retained without re-clustering or re-embedding, and the supplied log-normalized expression matrix was used directly. The 219 annotated clusters were grouped into 13 broad lineages for visualization. Expression of *arap3* was examined alongside that of the endothelial markers *kdrl*, *cdh5*, *fli1a*, *flt1*, *tek*, *tie1*, *pecam1a* and *cldn5b* using feature plots, a dot plot, stacked violin plots and a heatmap of mean expression scaled to z-scores for each gene. All analyses were performed using R version 4.4.3 and Seurat version 5.2.1. Custom analysis code is available at Zenodo (https://doi.org/10.5281/zenodo.20550202).

### EdU staining

The larvae were fixed in 4% PFA overnight at 4°C. The Click-iT EdU Alexa Fluor 647 Imaging Kit (Invitrogen, C10340) was used to assay the samples. EdU (100 μM) was injected into the CCV and ventricle of the brain in both wild type and *arap3* mutant at 32 hpf. The samples were then fixed at 72 hpf, and EdU staining was performed according to the manufacturer's protocol. Endothelial cells were labeled via anti-GFP antibody staining in the *Tg(kdrl:EGFP)* transgenic background.

### TUNEL assay

Larvae were fixed in 4% paraformaldehyde in PBS at 4°C overnight and assayed using the In Situ Cell Death Detection Kit, TMR Red (Roche), according to the manufacturer's instruction.

### Cell culture, transfection and western blot

HEK293T cells were obtained from the Cell Bank of the Chinese Academy of Sciences (Shanghai) and cultured in DMEM (Gibco, 11965092) supplemented with 10% fetal bovine serum (FBS; Gibco) and 1% penicillin–streptomycin at 37°C in a humidified atmosphere containing 5% CO_2_. Plasmid transfection was carried out using Lipofectamine 3000 Transfection Reagent (Invitrogen, L3000-001) in accordance with the manufacturer's instructions.

Cells were lysed in buffer containing 150 mM NaCl, 50 mM Tris-HCl (pH 8.0) and 1.0% Triton X-100, supplemented with 1 mM PMSF. Western blot analysis was performed using standard procedures. Briefly, proteins were resolved on 12% SDS–polyacrylamide gels (Bio-Rad) and transferred onto PVDF membranes. Membranes were blocked with 5% nonfat milk and incubated overnight at 4°C with the following primary antibodies: mouse Anti-HA (1:5000, Covance, E11FF01244) and rabbit anti-Myc (1:5000, Abcam, ab9106). After washing five times with blotting buffer, membranes were incubated for 2 h at room temperature with appropriate horseradish peroxidase-conjugated secondary antibodies: goat anti-mouse IgG HRP (1:5000, CST, 91196) or goat anti-rabbit IgG HRP (1:5000, CST, 7074). Protein signals were detected using SuperSignal West Pico Chemiluminescent Substrate (Thermo Fisher Scientific, 34577).

### Co-immunoprecipitation assay

For Arap3-Krit1 co-immunoprecipitation assays, HEK293T cells were transfected with 5 μg of *pcDNA3.1-Arap3-myc*, *pcDNA3.1-Krit1-2HA*, *pcDNA3.1-CCM2-Flag* and *pc DNA3.1-CCM2(F217A)-Flag* plasmids, either individually or in the indicated combinations. After 36 h of incubation, cells were harvested and lysed in non-denaturing lysis buffer [150 mM NaCl, 50 mM Tris-HCl (pH 7.4), 1% NP-40 and 1 mM EDTA] supplemented with protease inhibitors. One-third of each lysate was reserved as input and analyzed by western blot to assess protein expression levels. The remaining lysates were incubated with rabbit anti-Myc antibody (1:5000, Abcam, UK, ab9106) overnight at 4°C, followed by incubation with PureProteome Protein A/G Mix Magnetic Beads (Millipore, LSKMAGAG02) for 2 h at 4°C. Immunoprecipitated complexes were eluted with 2× SDS sample buffer, resolved on 12% SDS–polyacrylamide gels, and analyzed by immunoblotting with anti-HA and anti-Myc antibodies.

### mRNA synthesis and microinjection

Full-length coding sequences of *krit1*, *CA-rhoa* and *DN-rhoa* were amplified from zebrafish cDNA and cloned into the pCS2+ vector. Capped mRNA was synthesized using the mMESSAGE mMACHINE SP6 Transcription Kit (Ambion) according to the manufacturer's instructions. mRNA was microinjected into the yolk of zebrafish embryos at the one-cell stage using a microinjector.

### Heat shock induction

The *Tg(hsp70l:arap3-P2A-EGFP)* transgenic embryos were heat-shocked at 38.5°C for 30 min at the indicated stage, then incubated at 28.5°C until the indicated time points for phenotypic analysis.

### Active GTP-RHOA pulldown assay

Wild-type and *arap3^CRISPR^* mutant zebrafish embryos at 54 hpf were homogenized for total protein extraction, and active RhoA and total RhoA levels were determined using a commercial assay kit according to the manufacturer's instructions (RhoA Activation Assay Biochem Kit, Cytoskeleton, BK036).

### Rockout treatment

For pharmacological inhibition of ROCK signaling, dechorionated zebrafish embryos were treated with Rockout (MCE, 7272-84-6) at 10 μM from 30 to 48 hpf and subsequently maintained in 5 μM Rockout from 48 to 72 hpf. Embryos were incubated in embryo medium containing the indicated concentrations of Rockout, and control embryos were treated with equivalent concentrations of vehicle.

### Quantification and statistical analysis

All statistical analyses were performed using GraphPad Prism (GraphPad Software, USA). Data are presented as mean±s.d. or as box plots with min-to-max whiskers, as indicated in the figure legends. Embryos were obtained from multiple independent crosses. For *arap3^CRISPR^* mutant analyses, phenotyping was performed prior to genotyping, and the analyses were therefore genotype blinded. In other experiments, investigators were not blinded to group allocation during data collection or analysis. Siblings were randomly assigned to treatment groups where applicable. Each experiment was independently repeated at least twice, and the exact sample size (*n*) is provided in the figure legends. Statistical significance was assessed using one-way ANOVA followed by Holm–Šidák's multiple-comparisons test, the chi-square test or a two-tailed unpaired Student's *t*-test, as appropriate. *P*<0.05 was considered statistically significant. No data were excluded from the analyses.

Quantification of vascular length and branching was performed using the AngioTool software (National Cancer Institute, NIH). Fluorescent vascular images were analyzed using identical acquisition and processing parameters between experimental groups. Total vessel length and number of vessel junctions/branch points were quantified from the selected vascular regions.

Western blot band intensities were quantified using ImageJ/Fiji software (NIH). The signal intensity of each target protein was normalized to the corresponding loading control, and relative protein levels were calculated from at least three independent experiments.

### AI tool use declaration

During the preparation of this work, we used ChatGPT to summarize the current state of research about CCM pathogenesis. After using this tool, we reviewed and edited all content as needed, and take full responsibility for the content of the manuscript.

## Supplementary Material



10.1242/develop.205762_sup1 Supplementary information
