# Supplementary material for: Loss of arap3 drives a cerebral cavernous malformation-like phenotype in zebrafish via Krit1 interaction and RhoA dysregulation
Source: Development. 2026 Sep 21;153(18):dev205762. doi: 10.1242/dev.205762 (PMC13680834; doi:10.1242/dev.205762)
Supplement: Supplementary file 1 [file develop-153-205762-s1.pdf]

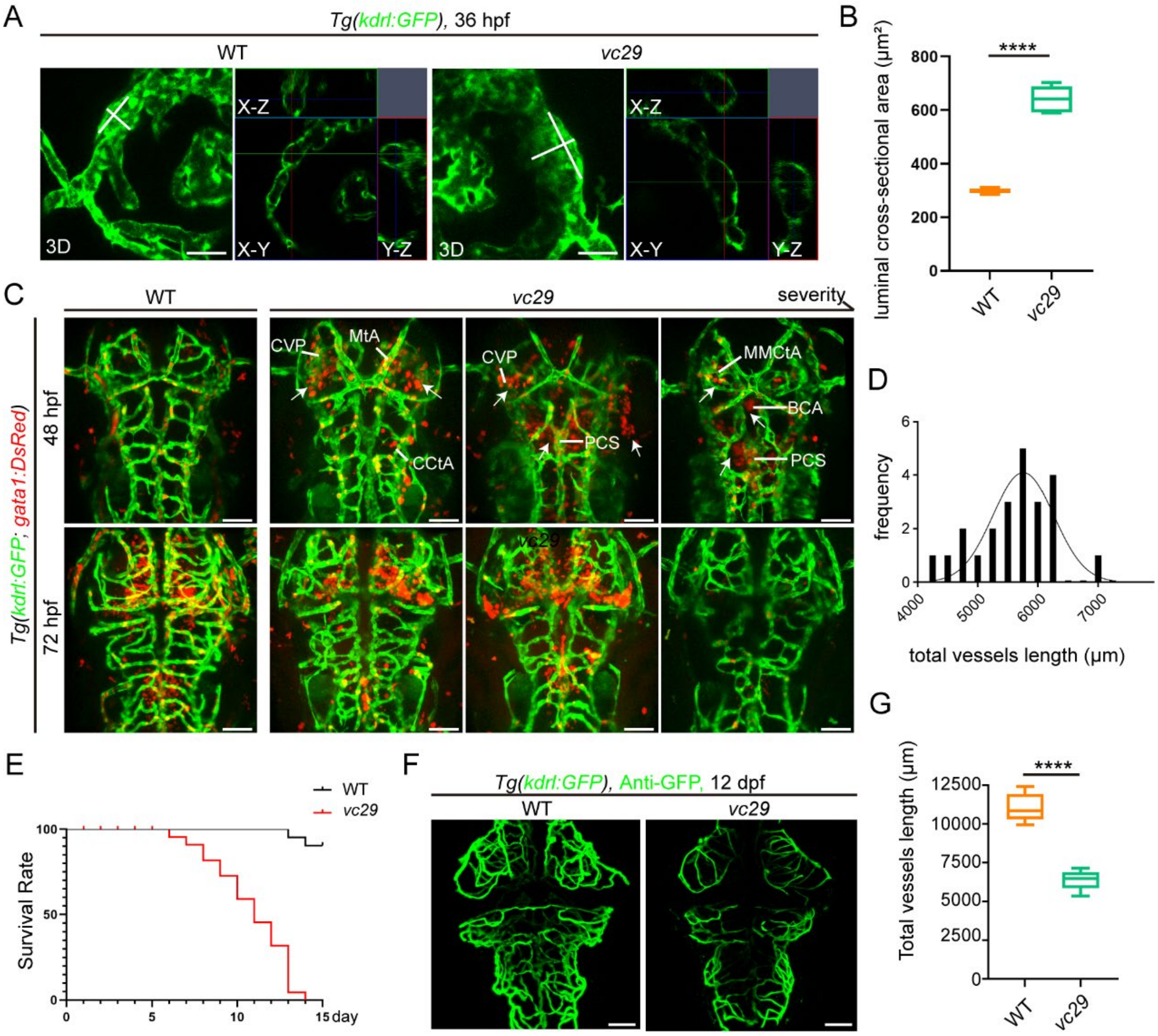

Fig. S1. Distribution of hemorrhage severity and lesion location in *vc29* mutants. A: Confocal images of the cerebrovasculature in WT and *vc29* mutant embryos carrying the Tg(*kdrl:GFP*) transgene at 36 hpf. Three-dimensional (3D) reconstruction and orthogonal projections (X–Y, X–Z, and Y–Z views) reveal abnormal vascular morphology and focal dilation in the *vc29* mutant compared with WT embryos. White lines indicate the corresponding vascular regions shown in orthogonal views. **B**: Quantification of vascular cross-sectional area/lumen area demonstrated significantly increased lumen size in *vc29* mutants (n=6) compared with WT embryos (n=6). **C**: Confocal images showing the hemorrhagic loci vary among individuals. White arrows indicate that the hemorrhagic sites were more likely to occur at branch points, and the corresponding vessels are labelled. **D**: Frequency distribution analysis of total vessel length at 72 hpf indicates an approximately normal distribution. **E**: Kaplan–Meier survival curves showing the survival of WT (n=20) and *arap3*^{CRISPR} mutant (n=20) during development. Survival was monitored

Development • Supplementary information

from 0 dpf to 15 dpf. **F**: Confocal images of the cerebrovasculature in WT and *vc29* mutant larvae at 12dpf. **G**: Quantification of total length of cerebral vessels in WT (n = 7), *vc29* mutant (n=6) and *arap3*^{CRISPR} mutant (n = 6) at 12 dpf. Data in panels B, G are presented as box plots. The center line indicates the median, the box indicates the interquartile range, and the whiskers indicate the minimum and maximum values. Data are presented as mean ± s.d, two-tailed unpaired t-test, asterisks indicate statistical significance: ****P < 0.0001. Scale bars, 50 µm (A, C, F). WT, wild type; hpf, hours post fertilization; CVP, choroidal vascular plexus; MtA, metencephalic artery; CCtA, cerebellar central artery; PCS, posterior (caudal) communicating segment; MMCtA, middle mesencephalic central artery.

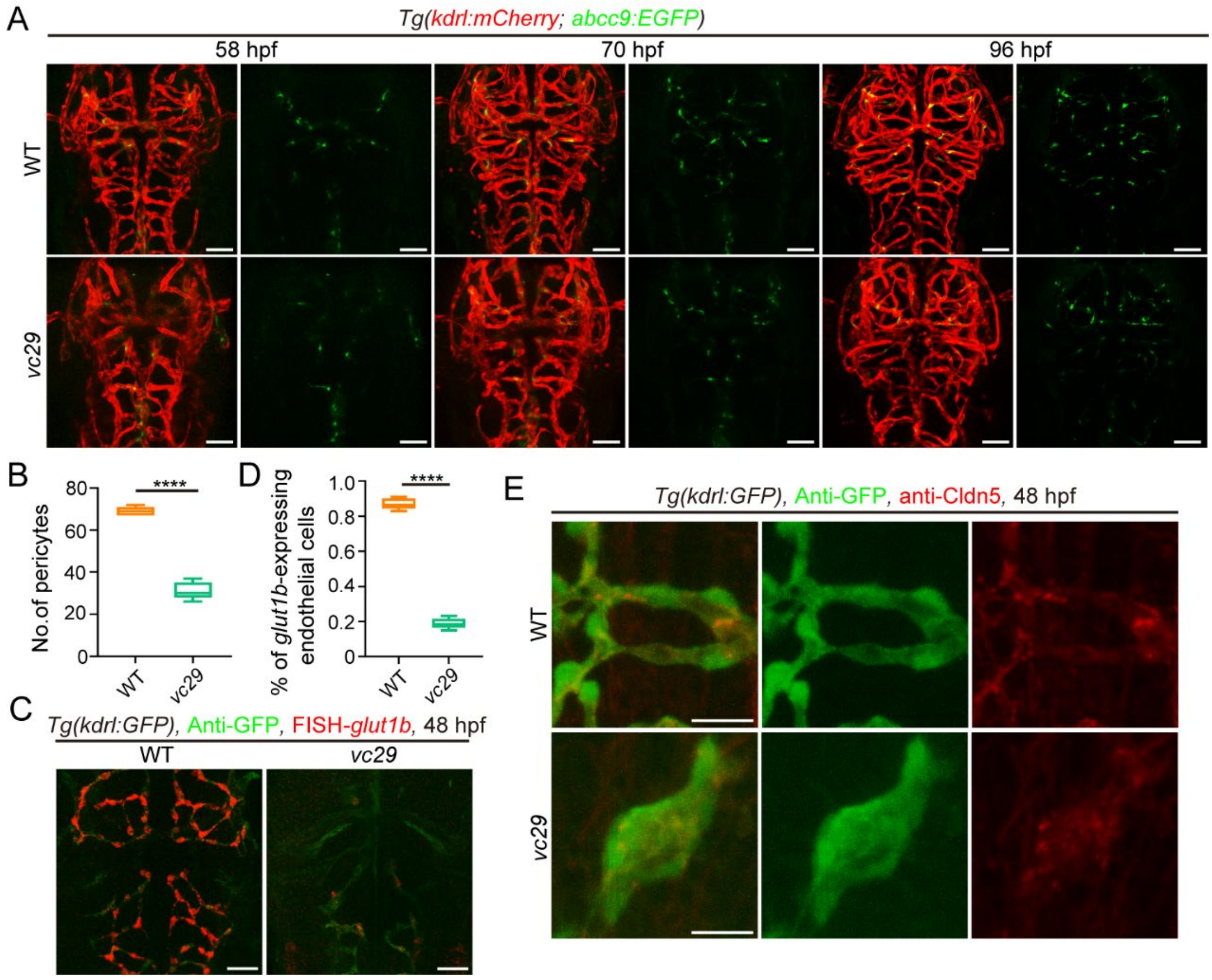

Fig. S2. Compromised blood-brain barrier integrity in *vc29* mutants. A: Time-lapse imaging of the cerebral vasculature and pericyte association in WT and *vc29* embryos from 58 hpf to 96 hpf in *Tg(kdrl:mCherry;abcc9:EGFP)*. **B:** Quantification of the number of the pericytes of WT (n = 7) and *vc29* (n = 8) at 96hpf. **C:** Fluorescent *in situ* hybridization (FISH) results showing the expression of *glut1b* at 48 hpf. **D:** Quantification of the percentage of *glut1b*-positive endothelial cells among kdrl-positive endothelial cells. **E:** Confocal images showing distribution of Cldn5. Data in panels B, D are presented as box plots. The center line indicates the median, the box indicates the interquartile range, and the whiskers indicate the minimum and maximum values. Data are presented as mean ± s.d, two-tailed unpaired t-test, asterisks indicate statistical significance: ****$P < 0.0001$. Scale bars, 50 μm. WT, wild type; hpf, hours post fertilization.

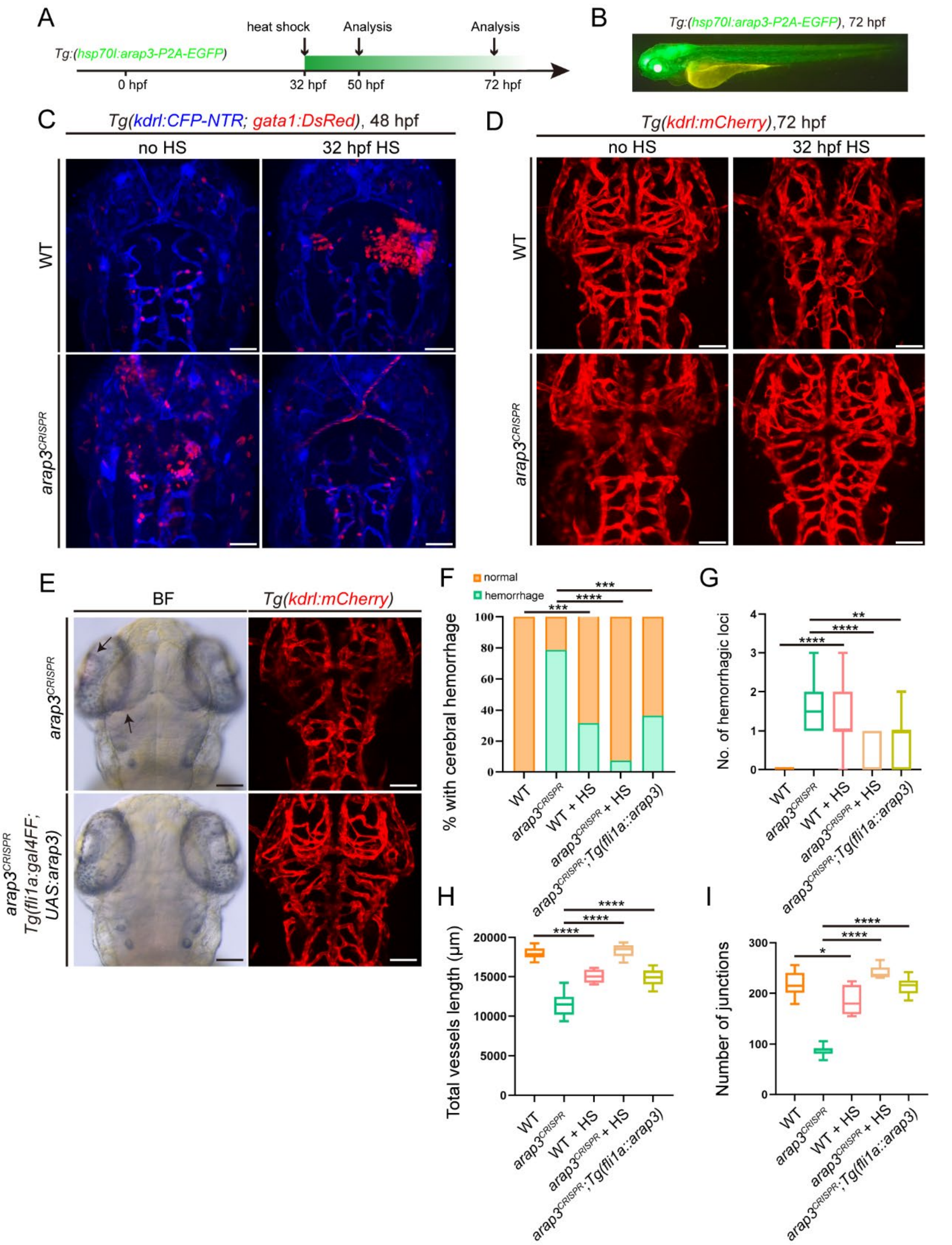

Fig. S3. Overexpression of *arap3* could partially rescue the *vc29* mutant phenotype. A: Schematic diagram of the heat shock regimen used for *Tg(hsp70l:arap3-P2A-EGFP)* embryos. Embryos were heat shocked at 32 hpf and analyzed at 50 and 72 hpf. **B**: Representative image of *Tg(hsp70l: arap3-P2A-EGFP)* embryo at 72 hpf showing EGFP expression after heat shock induction. **C**: Confocal images showing the cerebral vasculature in WT and *arap3*^{CRISPR} embryos of the *Tg(kdrl:CFP-NTR; gata1:DsRed)* line at 48 hpf with or without heat shock at 48 hpf.

Development • Supplementary information

D: Confocal images showing the cerebral vasculature in WT and *arap3*^{CRISPR} embryos of the *Tg(kdrl:mCherry)* line at 72 hpf with or without heat shock at 72 hpf. **E**: Confocal images showing *arap3*^{CRISPR} embryos and *arap3*^{CRISPR} with *Tg(fli1a:gal4FF; UAS:arap3)* in bright field and *Tg(kdrl:mCherry)* line at 72 hpf. **F:** Incidence of cerebral hemorrhage in WT embryos (n = 30), *arap3*^{CRISPR} embryos (n = 27), WT embryos after heat shock (n = 19), *arap3*^{CRISPR} embryos after heat shock (n = 27), and *arap3*^{CRISPR} with *Tg(fli1a:gal4FF; UAS:arap3)* (n=6) at 48 hpf. Statistical significance was assessed using the chi-square test**. G:** Quantification of the number of hemorrhagic foci of WT embryos (n = 30), *arap3*^{CRISPR} embryos (n = 27), WT embryos after heat shock (n = 19), *arap3*^{CRISPR} embryos after heat shock (n = 27), and *arap3*^{CRISPR} with *Tg(fli1a:gal4FF; UAS:arap3)* (n=6) at 48 hpf. **H**: Box plot showing the total cerebral vessel length in WT embryos (n = 7), *arap3*^{CRISPR} embryos (n = 14), WT embryos after heat shock (n = 11), *arap3*^{CRISPR} embryos after heat shock (n = 12), and *arap3*^{CRISPR} with *Tg(fli1a:gal4FF; UAS:arap3)* (n=6) at 72 hpf. The center line indicates the median, the box indicates the interquartile range, and the whiskers indicate the minimum and maximum values. **I**: Quantification of the number of vascular junctions in WT embryos (n = 7), *arap3*^{CRISPR} embryos (n = 14), WT embryos after heat shock (n = 11), *arap3*^{CRISPR} embryos after heat shock (n = 12), and *arap3*^{CRISPR} with *Tg(fli1a:gal4FF; UAS:arap3)* (n=6) at 72 hpf. Data are presented as mean ± s.d, statistical significance was assessed using one-way ANOVA followed by Holm–Šidák's multiple-comparisons test (G-I). Asterisks indicate statistical significance: *P < 0.05, **P < 0.01, ***P < 0.001, ****P < 0.0001. Scale bars, 50 μm (C, D, E). WT, wild type; hpf, hours post fertilization; HS, heat shock.

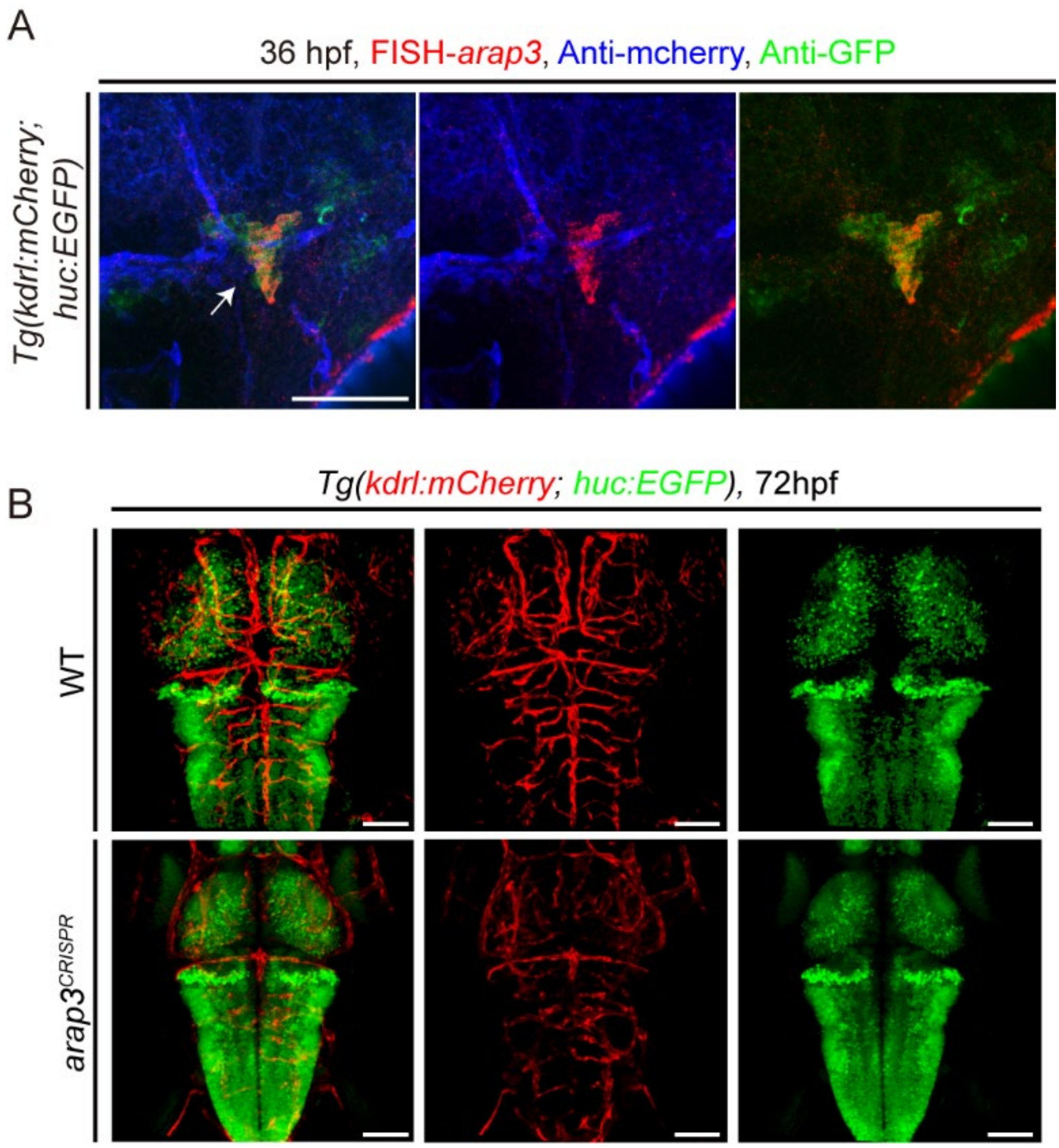

Fig. S4. Neuronal development is unaffected in *arap3* mutants. A: Fluorescent in situ hybridization for *arap3* combined with anti-mCherry and anti-GFP immunostaining in *Tg(kdrl:mCherry; huc:EGFP)* embryos at 36 hpf. *arap3* signal is shown in red. The white arrow indicates some neurons with enriched *arap3* expression. **B**: Confocal images showing the cerebral vasculature and neuronal pattern in WT and *arap3*^{CRISPR} embryos of the *Tg(kdrl:mCherry; huc:EGFP)* line at 72 hpf. No obvious defects in early neuronal development were observed in *arap3*^{CRISPR} embryos. Scale bars, 50 μm (A and B). WT, wild type; hpf, hours post fertilization; FISH, fluorescent *in situ* hybridization.

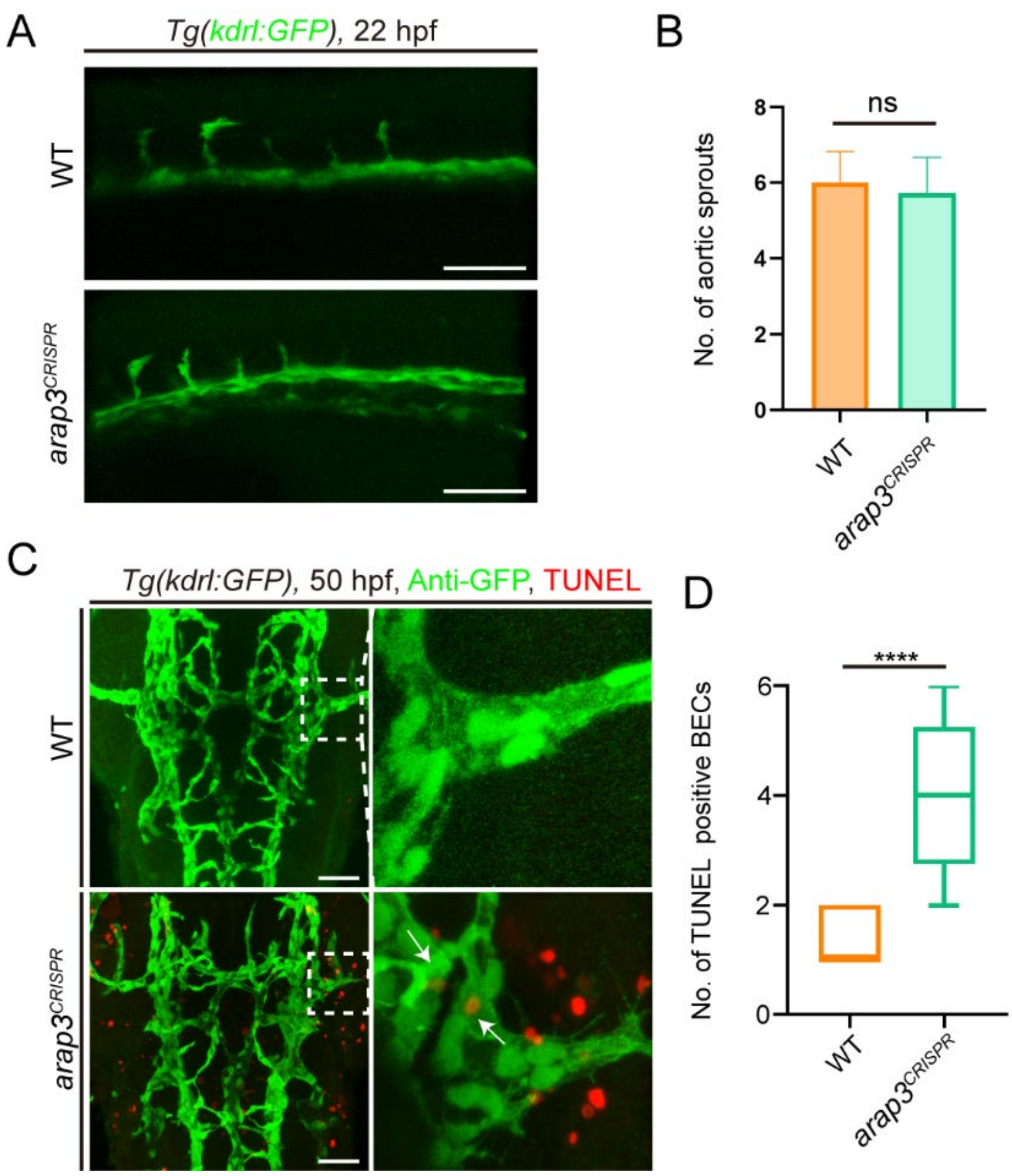

Fig. S5. Increased apoptosis in dilated cerebrovascular endothelium and preserved trunk angiogenesis in *arap3* mutants. A: Confocal images of trunk angiogenesis in WT and *arap3^{CRISPR}* embryos at 22 hpf. **B**: Quantification of the number of sprouts that emerge from the dorsal aorta of WT (n = 6) and *arap3^{CRISPR}* mutant (n = 6) at 22 hpf. **C**: Confocal images of cerebral vessels in WT and *arap3^{CRISPR}* embryos of the *Tg(kdrl:EGFP)* line at 50 hpf after anti-GFP immunostaining and TUNEL labeling. Right panels show higher-magnification views of the dashed-boxed regions. **D**: Quantification of the number of TUNEL-positive blood endothelial cells (BECs) in WT and *arap3^{CRISPR}* embryos. Data are presented as box plots (B, D). The center line indicates the median, the box indicates the interquartile range, and the whiskers indicate the minimum and maximum values. Data are presented as mean ± s.d, two-tailed unpaired t-test. Asterisks indicate statistical significance: ****$P < 0.0001$. Scale bars, 50 μm. WT, wild type; hpf, hours post fertilization; TUNEL, terminal deoxynucleotidyl transferase dUTP nick end labeling.

Development • Supplementary information

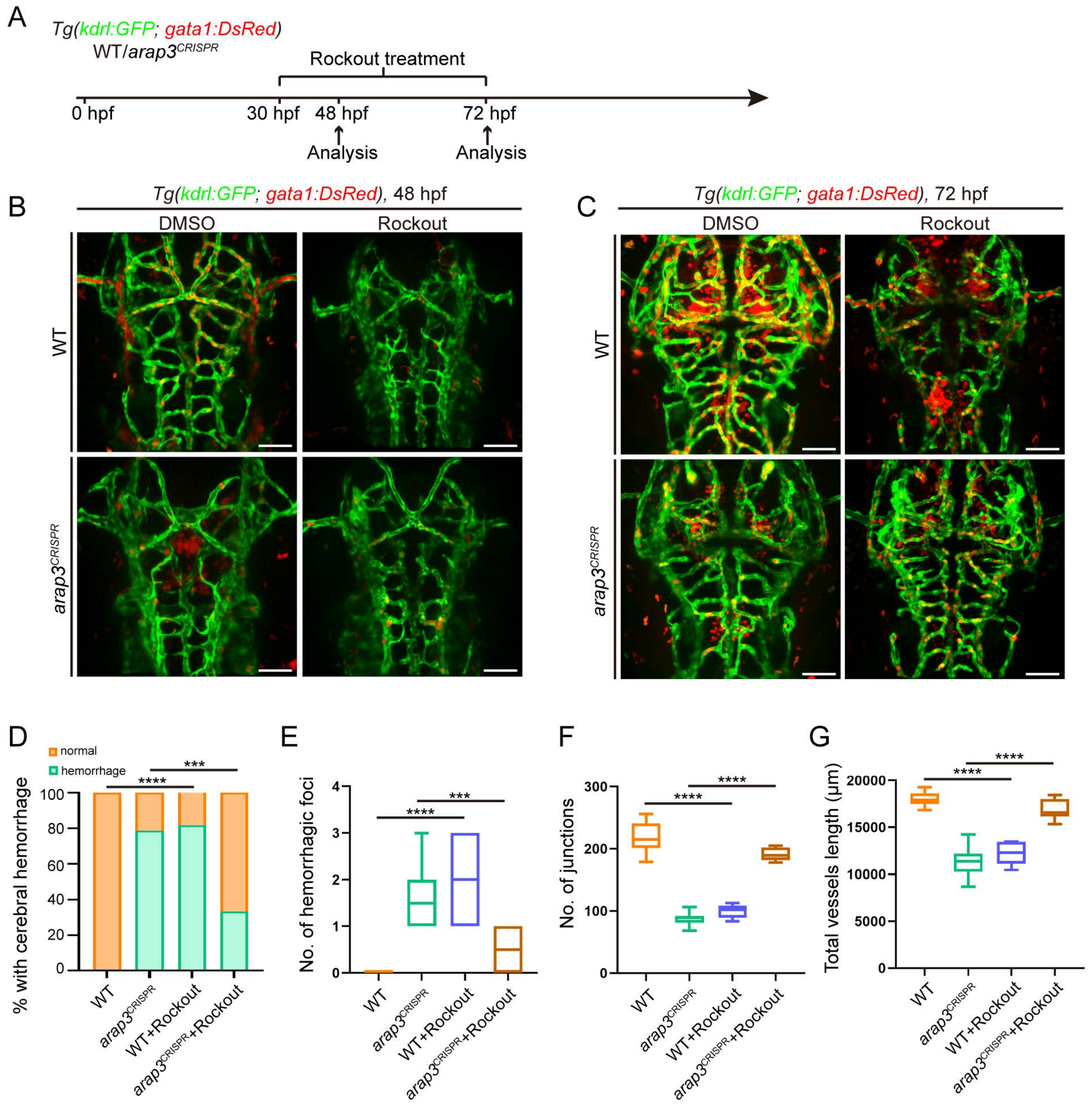

Fig. S6. ROCK inhibition provides robust rescue of cerebrovascular defects in *arap3* mutants. A: Schematic diagram of the Rockout treatment. WT and *arap3*^{CRISPR} embryos carrying the Tg(*kdrl:GFP; gata1:DsRed*) reporter were exposed to Rockout from 30 hpf onward (10 μM from 30–48 hpf and 5 μM from 48–72 hpf). Cerebrovascular phenotypes were analyzed at 48 hpf and 72 hpf. **B:** Confocal images showing the cerebral vasculature in WT and *arap3*^{CRISPR} embryos with Rockout/DMSO treatment at 48 hpf. **C:** Confocal images showing the cerebral vasculature in WT and *arap3*^{CRISPR} embryos with Rockout/DMSO treatment at 72 hpf. **D:** Incidence of cerebral hemorrhage in WT embryos (n = 10), *arap3*^{CRISPR} embryos (n = 12), WT embryos with Rockout (n = 10), *arap3*^{CRISPR} embryos with Rockout (n = 6) at 48 hpf. Statistical significance was assessed using the chi-square test**. E:** Quantification of the number of hemorrhagic foci of WT embryos (n = 10), *arap3*^{CRISPR} embryos (n = 12), WT embryos with Rockout (n = 10), *arap3*^{CRISPR} embryos with Rockout (n = 6) at 72 hpf. **F**: Quantification of the number of vascular junctions in WT

Development • Supplementary information

embryos (n = 10), *arap3*^{CRISPR} embryos (n = 12), WT embryos with Rockout (n = 10), *arap3*^{CRISPR} embryos with Rockout (n = 6) at 72 hpf. **G**: Box plot showing the total cerebral vessel length in WT embryos (n = 10), *arap3*^{CRISPR} embryos (n = 12), WT embryos with Rockout (n = 10), *arap3*^{CRISPR} embryos with Rockout (n = 6) at 72 hpf. Data in panels E-G are presented as box plots. The center line indicates the median, the box indicates the interquartile range, and the whiskers indicate the minimum and maximum values. Data are presented as mean ± s.d, statistical significance was assessed using one-way ANOVA followed by Holm–Šidák's multiple-comparisons test (E-G). Asterisks indicate statistical significance: ***P< 0.001, ****P < 0.0001. Scale bars, 50 μm. WT, wild type; hpf, hours post fertilization. Rockout, 3-(4-Pyridyl) indole.

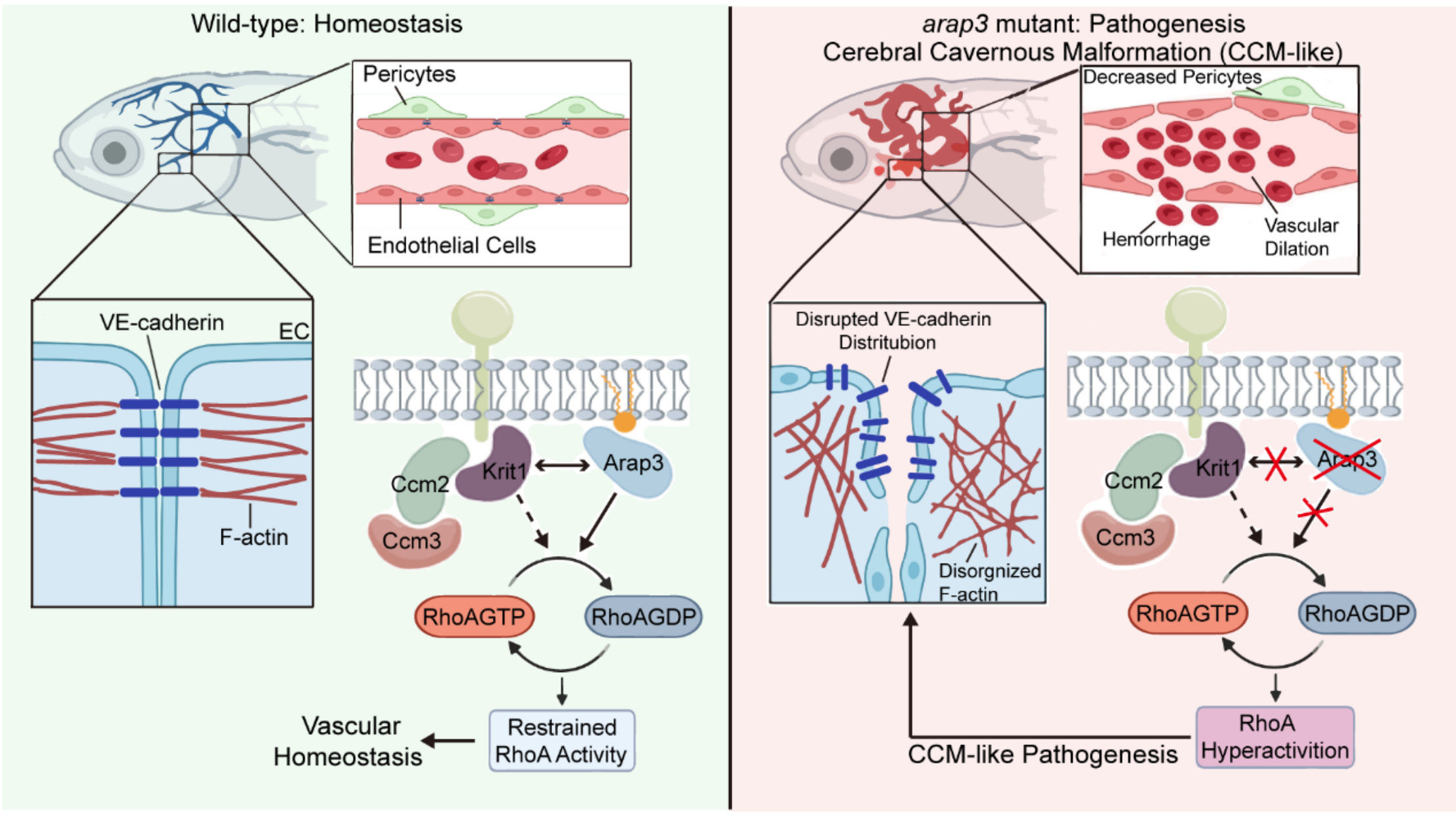

Fig. S7. Arap3 restricts RhoA activity to maintain cerebrovascular homeostasis and prevent CCM-like pathology. In wild-type embryos, Arap3 interacts with the CCM complex and functions as a RhoA GTPase-activating protein to limit RhoA signaling, thereby preserving VE-cadherin organization, endothelial junction integrity and F-actin cytoskeletal architecture. These coordinated effects preserve normal cerebrovascular morphology and vascular homeostasis. In *arap3* mutants, loss of Arap3 is associated with impaired CCM complex-related signaling and excessive RhoA activation, leading to disorganized VE-cadherin distribution, cytoskeletal disorganization, leaky endothelial junctions, lumen dilation and reduced pericyte coverage, ultimately resulting in CCM-like cerebrovascular lesions.

Development • Supplementary information
