# Supplementary material for: Loss of arap3 drives a cerebral cavernous malformation-like phenotype in zebrafish via Krit1 interaction and RhoA dysregulation
Source: Development. 2026 Sep 21;153(18):dev205762. doi: 10.1242/dev.205762 (PMC13680834; doi:10.1242/dev.205762)
Supplement: Supplementary file 2 [file develop-153-205762_review_history.pdf]

Loss of *arap3* drives a cerebral cavernous malformation-like phenotype in zebrafish via Krit1 interaction and RhoA dysregulation

Di Yang, Zewen Tao, Si Chen, Hongcheng Wang, Lingfei Luo and Jingying Chen
DOI: 10.1242/dev.205762

Editor: Kenneth Poss

Review timeline

Original submission:	23 April 2026
Editorial decision:	24 May 2026
First revision received:	15 July 2026
Editorial decision:	14 August 2026
Second revision received:	20 August 2026
Accepted:	23 August 2026

Original submission

First decision letter

MS ID#: dev.205762

MS TITLE: Loss of arap3 Drives a Cerebral Cavernous Malformation-Like Phenotype in Zebrafish via Krit1 Interaction and RhoA Dysregulation

AUTHORS: Di Yang, Zewen Tao, Si Chen, Hongcheng Wang, Lingfei Luo and Jingying Chen

Dear Dr Chen,

I have now received all the referees' reports on the above manuscript, and have reached a decision. The referees' comments are appended below.

As you will see, the referees express considerable interest in your work, but have some significant criticisms and recommend a substantial revision of your manuscript before we can consider publication. If you are able to revise the manuscript along the lines suggested, which may involve further experiments, I will be happy to receive a revised version of the manuscript. Your revised paper will be re-reviewed by one or more of the original referees, and acceptance of your manuscript will depend on your addressing satisfactorily the reviewers' major concerns. Please also note that Development will normally permit only one round of major revision. If it would be helpful, you are welcome to contact us to discuss your revision in greater detail. Please send us a point-by-point response indicating your plans for addressing the referees’ comments, and we will look over this and provide further guidance.

Please attend to all of the reviewers' comments and ensure that you upload both a 'clean' version of your Word file, along with a highlighted version clearly showing where you have made changes in the revised manuscript. Please avoid using 'Tracked changes' in Word files as these are lost in PDF conversion. I should be grateful if you would also provide a point-by-point response detailing how you have dealt with the points raised by the reviewers in the 'Response to Reviewers' box. If you do not agree with any of their criticisms or suggestions please explain clearly why this is so.

Reviewer 1

© 2026. Published by The Company of Biologists under the terms of the Creative Commons Attribution License (https://creativecommons.org/licenses/by/4.0/).

SUMMARY OF THE ADVANCE MADE IN THIS PAPER AND ITS POTENTIAL SIGNIFICANCE TO THE FIELD

Yang D. et al., report that arap3 deficiency induces cerebral cavernous malformation (CCM)-like phenotype in zebrafish. The authors identified arap3 as a causative gene for CCM-like blood vessel abnormalities by classical forward genetic screening using zebrafish. They tried to confirm that arap3 mutants exhibit CCM-like abnormalities in an endothelial cell autonomous manner. They further examined the association of arap3 with krit1 (CCM1) and investigated the role of RhoGAP in CCM-like phenotype by overexpressing constitutive active (CA)-rhoa and dominant negative (DN)-rhoa. Classical identification of arap3 as a causative gene for CCM-like phenotype by forward genetics is reliable. In addition, analyses of arap3 crispants support the relevance of arap3 deficiency to cerebral blood vessel abnormalities including dilation and hemorrhage in zebrafish embryos. However, the relationship between arap3 and CCM and the involvement of RhoA in vessel abnormalities are preliminary to support the authors' conclusions.

SUGGESTIONS TO AUTHORS

Major points

1. The physical and functional interaction of Krit1 and arap3 is not well supported by the authors' methods and data.
The proof of interaction between krit1 and arap3 is too preliminary. Binding of krit1 to CCM2 should be shown in the same co-immunoprecipitation (co-IP) assay using a positive control and a negative control. Mutated CCM2 that cannot bind to Krit1 should be used as a negative control (Fisher OS et al. J Biol. Chem. 290; 2842-2853, 2015). These fundamental approaches are essential to do co-IP to confirm the authors' methods and reliability of their data. Other krit1-binding molecules might be used to prove the authors' methods. Ideally, co-IP of endogenous molecules is the best as the authors describe in the limitation. To suggest that arap3 is in the CCM-regulated signaling cascade, they need to show the clear and reliable data for this interaction.
arap3 deficiency was rescued by overexpression of krit1 (Fig. 5D and 5E). Do the authors claim that arap3 is upstream of krit1? Given the hypothesis described in Fig. S6, krit1 unlikely rescues the phenotype in arap3 crispants. Do they claim that the biding of arap3 to krit1 inhibits krit1-dependent other GAP for RhoA? Or do they claim that krit1 regulates RhoGAP activity of arap3? These should be clarified by additional experiments.

2. Endogenous expression of arap3 should be examined using knock-in zebrafish. It is now easy to insert fluorescent protein that can be inserted into the last exon. At least BAC of arap3 clone can be used to examine its expression in endothelial cells. The quality of in situ hybridization data is low. In Fig. S4A, expression of arap3 cannot be detected in endothelial cells labeled with blue, which seems inconsistent with the authors' claim.

3. To show the endothelial cell autonomous role of arap3 and to prove the arap3-dependent rhoa regulation, either CA-rhoa or DN-rhoa should be expressed specifically in endothelial cells. The rescue experiment using Tg(hsp70l:arap3-P2A-EGFP) is not enough to show the importance of arap3's function. To confirm that the RhoGAP activity of arap3 is required for rescue, the authors can examine the effectiveness of rescue by overexpressing arap3 without either rhoGAP activity (point mutation) or arfGAP.

4. There might be misinterpretation of data.

(1) Red blood cells are easily observed in trunk vessels and caudal vein plexus of wild type embryos, whereas red blood cells are less in vc29 (Fig. 1F), indicating that blood circulation is impaired already at 48 hpf. The authors mention that 'Notably, trunk vascular development remained completely unaffected in vc29 mutant' (line 103-104). This description is inconsistent with the results of Fig. 1F. If blood cells decreased, blood vessel diameter must decrease.

(2) In dilated vessels, endothelial cell proliferation was demonstrated by the increased number of EdU-positive cells (Fig, 4E and 4F). However, the authors describe "reduced endothelial cell proliferation" (line 187). In the dilated vessels of arap3 crispants, endothelial cell number is increased.

(3) Expression of arap3 in arap3 crispants rescued the phenotype (hemorrhage) in Fig. S3. It is strange that although expression of arap3 in wild type induced hemorrhage, expression of arap3 rescued the hemorrhage in the crispants. In the lesions where arap3 deficiency caused hemorrhage, the phenotype might be rescued. Other regions where arap3 deficiency did not affect the

© 2026. Published by The Company of Biologists under the terms of the Creative Commons Attribution License (https://creativecommons.org/licenses/by/4.0/).

phenotype, hemorrhage should be present. When arap3 is overexpressed in the crispants, % with cerebral hemorrhage in arap3 crispants should be equal or greater than WT+HS, although it might be less than crispants. The actual data might be true as presented in Fig. S3E.

Minor problems

- To show the effect of GTP-bound RhoA on endothelial cell morphology, GTP-bound RhoA should be analyzed using the endothelial cell lysates instead of total lysates of embryos. Endothelial cells can be collected by cell sortingd.
- The authors' claim does not account for detachment of pericytes. Why are pericytes detached from cerebral endothelial cells?
- The authors use 'hemorrhage' and haemorrhage'.
- In the introduction section, the authors should not describe their present detailed data. The sentences that should be in result section are in the introduction. Previous findings of arap3 should be introduced.
- In the figure legend of Fig. 4D, Cdh5 on the endothelial cell surface is examined. What is cell surface Cdh5? Do they want to state the intercellular Cdh5?
- Amino acid number should be mentioned in Fig. 6B. Is Val of CA 14? Is Asn of DN 17? The amino acid number should be inserted as Fig. S2C.
- NTR is not explained. Zebrafish researchers might understand NTR. This should be spelled out. Please check other abbreviations.
- There are several papers that report Krit1-dependent RhoA signal. The authors should mention the potential regulation by arap3 and other RhoA-regulating molecules.
- Did the authors examine the effect of overexpression of CA-rhoa and DN-rhoa on other blood vessels? To show cell autonomous regulation by arap3 and rhoa in endothelial cells, they had better consider other methods instead of general expression of CA-rhoa and DN-rhoa.
- Did they also examine the effect of overexpression of arap3 on other blood vessels? They should show the data of other blood vessels. Other groups have already shown the importance of arap3 in lymphatic vessels (Kartopawiro J. et al.)
- The authors should use either Cdh5 (Fig. 4D) or VE-cadherin (Fig. S6) in the text and figures consistently.
- TUNEL-positive cells do not seem GFP-positive endothelial cells (Fig. S5), although an increase of number of TUNEL-positive cells is obvious. What are BECs (Fig. S5B)? Abbreviation should be spelled out when it is first used in the text or legends.
- Lumen dilatation is different from vessel enlargement. If the authors want to claim the lumen dilatation, cross section of vessels should be quantified.
- How did the authors measure the total vessel length? No description in the methods section.
- How did the author examine the abnormality of polarity? This is unclear in the present manuscript.

Reviewer 2

In this study, Yang and colleagues identified arap3 as a novel regulator of cerebrovascular development and demonstrated that loss of arap3 induces a CCM-like pathological phenotype. The authors showed that arap3 physically interacts with Krit1 and negatively regulates RhoA activity, thereby maintaining cerebrovascular stability. Overall, this study provides important insights into the mechanisms underlying cerebrovascular homeostasis. However, addressing the following points would further improve the clarity and impact of the manuscript.

1. The authors mainly characterized early embryonic cerebrovascular phenotypes (24-72 hpf). It would be valuable to quantify the survival rates of arap3 mutants and further examine cerebrovascular phenotypes at later developmental stages and in adult fish. These analyses would provide a more comprehensive understanding of the pathological consequences of arap3 deficiency.
2. Although the current data demonstrate vascular dilation and hemorrhage, direct evidence for blood-brain barrier (BBB) dysfunction is still lacking. The authors should consider performing whole-mount immunostaining or in vivo imaging using BBB-specific markers (e.g., glut1b and cldn5a) in both wild-type and arap3 mutant embryos to evaluate BBB integrity and vascular permeability. These experiments would strengthen the relevance of the phenotype to human CCM pathology.
3. The authors demonstrated a physical interaction between Arap3 and Krit1 by co-immunoprecipitation (Co-IP). However, the evidence for their spatial co-localization is missing.

© 2026. Published by The Company of Biologists under the terms of the Creative Commons Attribution License (https://creativecommons.org/licenses/by/4.0/).

Immunostaining experiments showing the co-localization of Arap3 and Krit1 would further support their functional interaction in vivo.
4. Since RhoA mainly exerts its effects through the downstream kinase ROCK during CCM pathogenesis, the authors should consider performing pharmacological rescue experiments using a specific ROCK inhibitor in arap3 mutants. Quantification of cerebrovascular dilation, hemorrhage, and endothelial integrity after treatment would help determine whether ROCK is the major downstream effector mediating the CCM-like phenotype.
5. In Fig. 3A, the region indicated by the white dashed box does not fully correspond to the magnified image. Please adjust the labeling to ensure consistency.
6. There are inconsistencies between the figure panels and legends in Fig. 3B. The legend states that embryos were analyzed at 36 hpf, whereas the figure includes both 24 and 36 hpf images. Similarly, another panel is labeled as 48 hpf only, while the figure actually contains both 36 and 48 hpf images. Please revise the figure legends accordingly.
7. In Fig. 4C, the Co-IP experiment should include detection of immunoprecipitated Myc-Arap3 to confirm comparable pull-down efficiency among samples.
8. Please correct several typographical errors in the figure legends, such as "showng" → "showing" and "haemorhage" → "hemorrhage".
9. In the Discussion section, the authors should further compare the arap3 mutant phenotype with previously reported zebrafish and mouse CCM models to better highlight the similarities, differences, and potential significance of this model.

Reviewer 3

SUMMARY OF THE ADVANCE MADE IN THIS PAPER AND ITS POTENTIAL SIGNIFICANCE TO THE FIELD

In this paper, Yang et al investigate the role of ARAP3 in the cerebral vasculature, using zebrafish as a model. This is a solid study indicating a relatively novel role for ARAP3 in the vasculature alongside a well-supported mechanism. It would be improved by greater consistency in the assessments of zebrafish mutant phenotype, alongside more quantifications and statistical analyses.

SUGGESTIONS TO AUTHORS

1. Line 156-157 potentially overstates effect of arap3 gRNA+fli1a:dCas9 on haemorrhage. Figure 3D indicates approximately 30% of these fish experience haemorrhage compared to 80% in the vc29 and arap3CRISPR mutants model. This is not therefore a full recapitulation. It would also be desirable to see more images from these EC-specific zebrafish, similar to that shown in Figure 1 for the vc29 model.
2. The Figures needs to be more consistent in how phenotype/haemorrhage is reported in each of the different fish mutants. For example, Figure 2 does not indicate the % of arap3CRISPR mutants that demonstrate haemorrhage, although this is shown in a supplement; the rescue in Figure 5 D-E should be assessed by the full range of assays utilized in Figure 1-2, similarly data in Figure 6 should be expanded.
3. There are publicly available scRNA-seq datasets for zebrafish at the timepoints of interest. Could the authors interrogate these for arap3 expression relative to brain/vascular markers, therefore confirming EC expression (and specificity within ECs) and detailing any other cell types expressing arap3?
4. The angiogenic sprouting defects shown in Figure 4B-C need quantification alongside n number and statistical verification of significance. Additional analysis should also be conducted on the trunk at the stage of early sprouting, to confirm the assertion in the discussion that these defects are specific to the cerebrovasculature.
5. Figure 6A requires quantification, n numbers and statistical analysis.
6. Figure 6C-G. I was confused by how the results of with CA-rhoa and DN-rhoa compare to those reported by Pillay 2022. Different names are used which makes comparison complicated, nor is it clear if the same vectors were used (text indicates yes, methods indicates no). Why the difference in haemorrhage between the two? It would be helpful to have this clarified, and for the whole range of phenotype assessments to be used consistently (e.g. why is % cerebral haemorrhage shown for CA rhoa but not DN rhoa; whilst number of hemorrhagic loci is shown for DN rhoa but not CA rhoa?). A discussion of the results here alongside Pillay 2022 should also be included in the discussion section.

© 2026. Published by The Company of Biologists under the terms of the Creative Commons Attribution License (https://creativecommons.org/licenses/by/4.0/).

First revision

Author response to reviewers' comments

Point by Point Response to the Reviewers

Response to the Reviewer #1

Major points

1. The physical and functional interaction of Krit1 and arap3 is not well supported by the authors' methods and data.

The proof of interaction between krit1 and arap3 is too preliminary. Binding of krit1 to CCM2 should be shown in the same co-immunoprecipitation (co-IP) assay using a positive control and a negative control. Mutated CCM2 that cannot bind to Krit1 should be used as a negative control (Fisher OS et al. J Biol. Chem. 290; 2842-2853, 2015). These fundamental approaches are essential to do co-IP to confirm the authors' methods and reliability of their data. Other krit1-binding molecules might be used to prove the authors' methods. Ideally, co-IP of endogenous molecules is the best as the authors describe in the limitation. To suggest that arap3 is in the CCM-regulated signaling cascade, they need to show the clear and reliable data for this interaction.

arap3 deficiency was rescued by overexpression of krit1 (Fig. 5D and 5E). Do the authors claim that arap3 is upstream of krit1? Given the hypothesis described in Fig. S6, krit1 unlikely rescues the phenotype in arap3 crispants. Do they claim that the biding of arap3 to krit1 inhibits krit1-dependent other GAP for RhoA? Or do they claim that krit1 regulates RhoGAP activity of arap3? These should be clarified by additional experiments.

Response to this point: We thank the reviewer for this constructive comment. We acknowledge the original co-IP data were preliminary and insufficient to confirm a robust, specific Krit1-ARAP3 interaction. In the revision, we validated our co-IP system with rigorous controls: Positive control: KRIT1 binding to wild-type CCM2; Negative control: CCM2-F217A, a PTB-domain mutant that abolishes KRIT1 binding (Fisher et al., J Biol Chem, 2015); Beads-only control to rule out non-specific pull-down. With this validated assay, we reproducibly detected ARAP3 in KRIT1 immunoprecipitates, with no signal in all negative controls. These results are now presented in Figure 6F and updated in the Results section.

Given the lack of validated antibodies for endogenous zebrafish Arap3/Krit1 co-IP, we have clearly stated this limitation and avoided overinterpreting the overexpression-based data.

We do not claim a simple linear upstream/downstream relationship between Arap3 and Krit1. The partial rescue by KRIT1 overexpression suggests that increased KRIT1 dosage compensates for *arap3* deficiency via a shared endothelial vascular stability pathway converging on junctional integrity and RhoA/ROCK regulation. We further supplemented pMLC-ATPase activity assays (Fig. 6L): *arap3* mutants exhibited elevated ATPase activity, which was attenuated by *krit1* mRNA co-injection, indicating KRIT1 rescues the hemorrhage phenotype by normalizing this dysregulation. We have revised the manuscript to describe ARAP3 as functionally associated with the CCM/KRIT1 pathway, rather than defining a strict linear epistatic relationship.

2. Endogenous expression of arap3 should be examined using knock-in zebrafish. It is now easy to insert fluorescent protein that can be inserted into the last exon. At least BAC of arap3 clone can be used to examine its expression in endothelial cells. The quality of in situ hybridization data is low. In Fig. S4A, expression of arap3 cannot be detected in endothelial cells labeled with blue, which seems inconsistent with the authors' claim.

© 2026. Published by The Company of Biologists under the terms of the Creative Commons Attribution License (https://creativecommons.org/licenses/by/4.0/).

Response to this point: We thank the reviewer for this valuable suggestion. We agree that endogenous knock-in fluorescent reporters or BAC-based *arap3* reporters are the gold standard for definitive in vivo expression profiling. However, generating these stable transgenic lines requires extensive time for construct engineering, founder screening, germline transmission and validation, which is not feasible within the current revision timeline.

To address concerns about *in situ* hybridization quality and endothelial expression, we have supplemented our analysis with two independent lines of evidence: 1. Fluorescence in situ hybridization (FISH) coupled with antibody staining (He et al., Nat Protoc, 2020) verifies clear *arap3* expression in vascular endothelial cells at single-cell resolution (Fig. 3B). Low-level *arap3* signal is also detected in a small subset of neurons (Fig. S4A), reflecting its non-endothelium-restricted expression pattern; 2. Reanalysis of publicly available zebrafish single-cell RNA-seq datasets at phenotype-relevant stages confirms the presence of *arap3* transcripts in endothelial clusters (marked by the canonical markers *kdrl*, *fli1a*, *flt4*, *cdh5*), consistent with our experimental findings (Fig. 3C, D). These data have been integrated into the revised manuscript to strengthen the evidence for endothelial expression of *arap3*.

3. To show the endothelial cell autonomous role of arap3 and to prove the arap3-dependent rhoa regulation, either CA-rhoa or DN-rhoa should be expressed specifically in endothelial cells. The rescue experiment using Tg(hsp70l:arap3-P2A-EGFP) is not enough to show the importance of arap3's function. To confirm that the RhoGAP activity of arap3 is required for rescue, the authors can examine the effectiveness of rescue by overexpressing arap3 without either rhoGAP activity (point mutation) or arfGAP.

Response to this point: To address the limitation of ubiquitous overexpression and verify the endothelial cell-autonomous function of *arap3* as well as its RhoA-dependent regulation, we generated transgenic lines for endothelial-specific overexpression of DN-RhoA and wild-type Arap3. In *arap3* mutants, endothelial-specific DN-RhoA expression partially rescued the vascular phenotype, directly confirming that *arap3* exerts its function through endothelial RhoA signaling (Fig. 5F-K). Endothelial-specific Arap3 overexpression also significantly rescued the mutant phenotype, demonstrating the functional importance of endothelial *arap3* (Fig. S3E-I). We agree that rescue assays with RhoGAP-dead or ArfGAP-dead Arap3 mutants are critical to validate the enzymatic requirement for Arap3 function. This important mechanistic experiment will be prioritized in our future studies, please see our Limitation.

4. There might be misinterpretation of data.
(1) Red blood cells are easily observed in trunk vessels and caudal vein plexus of wild type embryos, whereas red blood cells are less in vc29 (Fig. 1F), indicating that blood circulation is impaired already at 48 hpf. The authors mention that 'Notably, trunk vascular development remained completely unaffected in vc29 mutant' (line 103-104). This description is inconsistent with the results of Fig. 1F. If blood cells decreased, blood vessel diameter must decrease.

Response to this point: We apologize for the misunderstanding caused by the original image. The reduced red blood cells in trunk vessels in Fig.1F were an artefact induced by tricaine anaesthesia during imaging, rather than a defect in vascular development. We have replaced this image to eliminate misinterpretation, and confirm that trunk vascular development remains completely unaffected in *vc29* mutants at 48 hpf.

(2) In dilated vessels, endothelial cell proliferation was demonstrated by the increased number of EdU-positive cells (Fig, 4E and 4F). However, the authors describe "reduced endothelial cell proliferation" (line 187). In the dilated vessels of arap3 crispants, endothelial cell number is increased.

Response to this point: We apologize for the ambiguous description in the text. EdU-positive endothelial cells were quantified across the whole cerebral vasculature, showing an overall reduction in endothelial proliferation. However, locally dilated vascular lesions exhibited concentrated EdU signals (Fig. 4F), which stem from disrupted endothelial homeostasis and aberrant local proliferation that drives vascular dilation and hemorrhage, consistent with the

© 2026. Published by The Company of Biologists under the terms of the Creative Commons Attribution License (https://creativecommons.org/licenses/by/4.0/).

phenotype in Fig. 4B. We have revised the relevant text to clarify this distinction between global and local proliferation changes.

(3) Expression of arap3 in arap3 crispants rescued the phenotype (hemorrhage) in Fig. S3. It is strange that although expression of arap3 in wild type induced hemorrhage, expression of arap3 rescued the hemorrhage in the crispants. In the lesions where arap3 deficiency caused hemorrhage, the phenotype might be rescued. Other regions where arap3 deficiency did not affect the phenotype, hemorrhage should be present. When arap3 is overexpressed in the crispants, % with cerebral hemorrhage in arap3 crispants should be equal or greater than WT+HS, although it might be less than crispants. The actual data might be true as presented in Fig. S3E.

Response to this point: Cerebral hemorrhage sites are stochastic due to hemodynamic shear stress. Re-expression of *arap3* in *arap3* crispants restores Arap3 function globally across the entire cerebral vasculature, rather than acting only on pre-existing hemorrhagic lesions. In the mutant background with endogenous *arap3* deficiency, restored *arap3* expression returns to a physiologically functional range and corrects the vascular homeostasis defect. This globally reduces hemorrhage risk throughout the vasculature, instead of inducing additional hemorrhage in originally unaffected regions, which is consistent with the quantification in Fig. S3F.

Minor problems
To show the effect of GTP-bound RhoA on endothelial cell morphology, GTP-bound RhoA should be analyzed using the endothelial cell lysates instead of total lysates of embryos. Endothelial cells can be collected by cell sortingd.

Response to this point: We attempted to perform GTP-RhoA pull-down assays on FACS-sorted endothelial cells. However, the sorting procedure is time-consuming and difficult to maintain cell viability, which would alter the native GTP-bound state of RhoA and fail to reflect its in vivo activation status. This technical limitation remains challenging and has not been well resolved in the field.

The authors' claim does not account for detachment of pericytes. Why are pericytes detached from cerebral endothelial cells?

Response to this point: We performed long-term time-lapse live imaging and did not observe pericyte detachment from cerebral endothelial cells. The reduced pericyte number arises from impaired cerebral vascular maturation and blood-brain barrier development, which disrupts pericyte recruitment, as supported by Fig. S2A-E.

The authors use 'hemorrhage' and haemorrhage'.

Response to this point: Thank you for pointing this out. We have standardized the spelling uniformly throughout the entire manuscript.

In the introduction section, the authors should not describe their present detailed data. The sentences that should be in result section are in the introduction. Previous findings of arap3 should be introduced.

Response to this point: Thank you for your suggestion. We have revised the introduction: we removed detailed result descriptions that properly belong in the results section, and supplemented the introduction with previously published findings on *arap3*.

In the figure legend of Fig. 4D, Cdh5 on the endothelial cell surface is examined. What is cell surface Cdh5? Do they want to state the intercellular Cdh5?

Response to this point: Thank you for the correction. The description "cell surface Cdh5" was inaccurate. We have revised it to "junctional VE-cadherin" in the figure 4E legend.

Amino acid number should be mentioned in Fig. 6B. Is Val of CA 14? Is Asn of DN 17? The amino acid number should be inserted as Fig. S2C.

© 2026. Published by The Company of Biologists under the terms of the Creative Commons Attribution License (https://creativecommons.org/licenses/by/4.0/).

Response to this point: Thank you for the reminder. We have added the corresponding amino acid residue numbers in the schematic diagram of Fig. 6C, consistent with the annotation style in Fig. 2D.

NTR is not explained. Zebrafish researchers might understand NTR. This should be spelled out. Please check other abbreviations.
Response to this point: We have spelled out the full name of NTR at its first appearance in the figure legend, and checked all other abbreviations in the manuscript to ensure full definitions are provided on first mention.

There are several papers that report Krit1-dependent RhoA signal. The authors should mention the potential regulation by arap3 and other RhoA-regulating molecules.
Response to this point: Thank you for this valuable suggestion. We have added discussion of Krit1 and other RhoA regulatory molecules, and elaborated the potential regulatory relationship between Arap3 and other signaling pathways in the Discussion section.

Did the authors examine the effect of overexpression of CA-rhoa and DN-rhoa on other blood vessels? To show cell autonomous regulation by arap3 and rhoa in endothelial cells, they had better consider other methods instead of general expression of CA-rhoa and DN-rhoa.

Response to this point: To verify the endothelial cell-autonomous regulatory effect, we performed rescue experiments with endothelial-specific overexpression of *arap3* and *DN-rhoa*. The corresponding results are now presented in Fig. S3E and Fig. 5F.

Did they also examine the effect of overexpression of arap3 on other blood vessels? They should show the data of other blood vessels. Other groups have already shown the importance of arap3 in lymphatic vessels (Kartopawiro J. et al.)
Response to this point: We also examined lymphatic vessel development in the mutants. No developmental abnormality of the thoracic duct (TD) was detected at 5 dpf, which differs from the reported lymphatic phenotype in previous work (Kartopawiro J. et al.).

The authors should use either Cdh5 (Fig. 4D) or VE-cadherin (Fig. S6) in the text and figures consistently.

Response to this point: Thank you for pointing out this inconsistency. We have standardized the term to VE-cadherin uniformly across the full text and all figure legends in the revised manuscript.

TUNEL-positive cells do not seem GFP-positive endothelial cells (Fig. S5), although an increase of number of TUNEL-positive cells is obvious. What are BECs (Fig. S5B)? Abbreviation should be spelled out when it is first used in the text or legends.

Response to this point: We acknowledge that most TUNEL-positive signals do not co-localize with GFP-labeled endothelial cells. We have added arrows to mark the overlapping signals in Fig. S5C and revised the figure legend accordingly. The abbreviation "BEC" has been spelled out in full at its first appearance in both the text and figure legend

Lumen dilatation is different from vessel enlargement. If the authors want to claim the lumen dilatation, cross section of vessels should be quantified.

Response to this point: We have added cross-sectional images of cerebral vessels in Fig. S1A-B and performed quantitative analysis of lumen diameter to verify lumen dilatation.

How did the authors measure the total vessel length? No description in the methods section.
Response to this point: Total vessel length was quantified using AngioTool software. We have added the detailed description of this quantification method to the Materials and Methods section.

How did the author examine the abnormality of polarity? This is unclear in the present manuscript.

© 2026. Published by The Company of Biologists under the terms of the Creative Commons Attribution License (https://creativecommons.org/licenses/by/4.0/).

Response to this point: We evaluated cell polarity based on abnormal aggregation of Lifeact-labeled filamentous actin in mutant cerebral vessels. As established in prior work (Phng et al., Development 2013), proper filopodia formation and endothelial polarity rely on tightly regulated actin polymerization; dysregulated actin aggregation therefore indicates disrupted cell polarity during angiogenesis. We have clarified and refined this description in the revised manuscript.

Response to the Reviewer #2
1. The authors mainly characterized early embryonic cerebrovascular phenotypes (24-72 hpf). It would be valuable to quantify the survival rates of arap3 mutants and further examine cerebrovascular phenotypes at later developmental stages and in adult fish. These analyses would provide a more comprehensive understanding of the pathological consequences of arap3 deficiency.

Response to this point: We have quantified the survival rate of arap3 mutants. Most mutants die by 15 days post-fertilization (dpf) and cannot survive to adulthood; severely affected mutants show early lethality with pericardial edema. We also observed prominent cerebrovascular developmental abnormalities in mutants at 12 dpf (Fig. S1E-G).

2. Although the current data demonstrate vascular dilation and hemorrhage, direct evidence for blood-brain barrier (BBB) dysfunction is still lacking. The authors should consider performing whole-mount immunostaining or in vivo imaging using BBB-specific markers (e.g., glut1b and cldn5a) in both wild-type and arap3 mutant embryos to evaluate BBB integrity and vascular permeability. These experiments would strengthen the relevance of the phenotype to human CCM pathology.

Response to this point: We examined the expression of BBB-specific markers *glut1b* and Cldn5a. In *arap3* mutant cerebral vessels, *glut1b* expression was significantly downregulated, and the tight junction protein Cldn5 showed disorganized localization. Pericytes coverage on cerebral vessels was also markedly reduced in mutants (Fig. S2D-H). These data confirm BBB developmental defects and support that the arap3 mutant recapitulates CCM pathology.

3. The authors demonstrated a physical interaction between Arap3 and Krit1 by co-immunoprecipitation (Co-IP). However, the evidence for their spatial co-localization is missing. Immunostaining experiments showing the co-localization of Arap3 and Krit1 would further support their functional interaction in vivo.

Response to this point: We have added relevant data in Fig. 6G using fluorescence in situ hybridization (FISH) combined with antibody staining. The results demonstrate that both *arap3* and *krit1* are expressed in vascular endothelial cells *in vivo*, verifying their spatial co-localization in the vasculature.

4. Since RhoA mainly exerts its effects through the downstream kinase ROCK during CCM pathogenesis, the authors should consider performing pharmacological rescue experiments using a specific ROCK inhibitor in arap3 mutants. Quantification of cerebrovascular dilation, hemorrhage, and endothelial integrity after treatment would help determine whether ROCK is the major downstream effector mediating the CCM-like phenotype.

Response to this point: Thanks for this suggestion. We have completed ROCK inhibitor (Rockout) rescue experiments, and the results are presented in Fig. S6. Rockout treatment significantly rescued the CCM-like cerebrovascular phenotypes in *arap3* mutant larvae, confirming that ROCK is a key downstream effector of Arap3 in CCM pathogenesis

5. In Fig. 3A, the region indicated by the white dashed box does not fully correspond to the magnified image. Please adjust the labeling to ensure consistency.

Response to this point: Thank you for pointing this out. We have revised Fig. 3A to ensure the dashed box fully matches the magnified inset region.

6. There are inconsistencies between the figure panels and legends in Fig. 3B. The legend states that embryos were analyzed at 36 hpf, whereas the figure includes both 24 and 36 hpf images.

© 2026. Published by The Company of Biologists under the terms of the Creative Commons Attribution License (https://creativecommons.org/licenses/by/4.0/).

Similarly, another panel is labeled as 48 hpf only, while the figure actually contains both 36 and 48 hpf images. Please revise the figure legends accordingly.

Response to this point: Thank you for the correction. We have revised the figure legends of Fig. 3 to accurately reflect the time points shown in each panel.

7. In Fig. 4C, the Co-IP experiment should include detection of immunoprecipitated Myc-Arap3 to confirm comparable pull-down efficiency among samples.

Response to this point: In the revision, we validated our co-IP system with rigorous controls: Positive control: KRIT1 binding to wild-type CCM2; Negative control: CCM2-F217A, a PTB-domain mutant that abolishes KRIT1 binding (Fisher et al., J Biol Chem, 2015); Beads-only control to rule out non-specific pull-down. With this validated assay, we reproducibly detected ARAP3 in KRIT1 immunoprecipitates, with no signal in all negative controls. These results are now presented in Figure 6F and updated in the Results section.

8. Please correct several typographical errors in the figure legends, such as "showng" → "showing" and "haemorhage" → "hemorrhage".

Response to this point: Thank you for pointing out these typos. We have corrected all spelling errors in the figure legends, including the two noted above.

9. In the Discussion section, the authors should further compare the arap3 mutant phenotype with previously reported zebrafish and mouse CCM models to better highlight the similarities, differences, and potential significance of this model

Response to this point: Thank you for this valuable suggestion. We have expanded the Discussion section to include a systematic comparison of our *arap3* model with established zebrafish and mouse CCM models, to clarify the similarities, differences and translational significance of our work.

Response to the Reviewer #3

1. Line 156-157 potentially overstates effect of arap3 gRNA+fli1a:dCas9 on haemorrhage. Figure 3D indicates approximately 30% of these fish experience haemorrhage compared to 80% in the vc29 and arap3CRISPR mutants model. This is not therefore a full recapitulation. It would also be desirable to see more images from these EC-specific zebrafish, similar to that shown in Figure 1 for the vc29 model.

Response to this point: We agree that the phrase "a full recapitulation" was an overstatement. We have revised the text to "was sufficient to recapitulate" to more accurately describe our findings. The EC-specific *arap3* knockdown was generated via 1-cell stage microinjection of dCas9 plasmid, gRNA and tol2 mRNA, which produces mosaic knockdown with variable efficiency due to technical limitations. This accounts for the lower hemorrhage rate compared with stable germline mutants. To fully characterize the phenotype, we have quantified multiple readouts including number of hemorrhagic foci, total vessel length, vessel branch count and circulation impairment rate (Fig. 3F-J).

2. The Figures needs to be more consistent in how phenotype/haemorrhage is reported in each of the different fish mutants. For example, Figure 2 does not indicate the % of arap3CRISPR mutants that demonstrate haemorrhage, although this is shown in a supplement; the rescue in Figure 5 D-E should be assessed by the full range of assays utilized in Figure 1-2, similarly data in Figure 6 should be expanded.

Response to this point: We have added unified quantitative analyses as suggested, including phenotypic statistics in Fig. 5G-K and Fig. 6B-E, I-K, to ensure consistent assessment metrics across all figures.

3. There are publicly available scRNA-seq datasets for zebrafish at the timepoints of interest. Could the authors interrogate these for arap3 expression relative to brain/vascular markers, therefore

© 2026. Published by The Company of Biologists under the terms of the Creative Commons Attribution License (https://creativecommons.org/licenses/by/4.0/).

confirming EC expression (and specificity within ECs) and detailing any other cell types expressing arap3?

Response to this point: Thank you for this suggestion. We analyzed the published zebrafish scRNA-seq dataset (Farnsworth et al., 2020) and found that *arap3* is predominantly enriched in endothelial cells, with lower expression observed in neurons. This is consistent with our FISH results, and the relevant analysis data are now presented in Fig. 3C and 3D.

4. The angiogenic sprouting defects shown in Figure 4B-C need quantification alongside n number and statistical verification of significance. Additional analysis should also be conducted on the trunk at the stage of early sprouting, to confirm the assertion in the discussion that these defects are specific to the cerebrovasculature.

Response to this point: We have supplemented the quantification, sample numbers and statistical analysis for sprouting defects as suggested (Fig. 4C). We also added analysis of trunk angiogenic sprouting at early stages to validate the cerebrovascular specificity of the phenotype (Fig. S5A–B).

5. Figure 6A requires quantification, n numbers and statistical analysis.

Response to this point: We have added the corresponding quantification, n numbers and statistical analysis, now shown in Fig. 5B.

6. Figure 6C-G. I was confused by how the results of with CA-rhoa and DN-rhoa compare to those reported by Pillay 2022. Different names are used which makes comparison complicated, nor is it clear if the same vectors were used (text indicates yes, methods indicates no). Why the difference in haemorrhage between the two? It would be helpful to have this clarified, and for the whole range of phenotype assessments to be used consistently (e.g. why is % cerebral haemorrhage shown for CA rhoa but not DN rhoa; whilst number of hemorrhagic loci is shown for DN rhoa but not CA rhoa?). A discussion of the results here alongside Pillay 2022 should also be included in the discussion section.

Response to this point: We apologize for the confusion. We used the same CA-rhoa and DN-rhoa sequences reported in Pillay et al., 2022, but adopted a different delivery method: Pillay et al. used stable transgenic lines, while we performed microinjection of *in vitro* transcribed mRNA cloned into the *pCS2* vector. The phenotypic trend is consistent with the previous report: both hyperactivation and inhibition of RhoA activity cause hemorrhage. We have unified the phenotypic quantification for all mRNA injection groups (Fig. 5G-K) and added a detailed comparison with Pillay 2022 in the Discussion section.

Second decision letter

MS ID#: dev.205762R1

MS TITLE: Loss of arap3 Drives a Cerebral Cavernous Malformation-Like Phenotype in Zebrafish via Krit1 Interaction and RhoA Dysregulation

AUTHORS: Di Yang, Zewen Tao, Si Chen, Hongcheng Wang, Lingfei Luo and Jingying Chen

Dear Dr Chen,

I have now received all the referees reports on the above manuscript, and have reached a decision. The referees' comments are appended below.

The overall evaluation is positive and we would like to publish a revised manuscript in Development. Please attend to all of the reviewers' comments in your revised manuscript and detail

© 2026. Published by The Company of Biologists under the terms of the Creative Commons Attribution License (https://creativecommons.org/licenses/by/4.0/).

them in your point-by-point response. Note that there are minor requests by Reviewer 1, which I hope you will be able to address without taking too much time and without a need for me to send back to the reviewer. If you do not agree with any of their criticisms or suggestions explain clearly why this is so. If it would be helpful, you are welcome to contact us to discuss your revision in greater detail. Please send us a point-by-point response indicating your plans for addressing the referees' comments, and we will look over this and provide further guidance.

Reviewer 1

SUMMARY OF THE ADVANCE MADE IN THIS PAPER AND ITS POTENTIAL SIGNIFICANCE TO THE FIELD

This manuscript demonstrates the potential importance of Arap3 in Cerebral Cavernous Malformation-like pathology in zebrafish. Arap3 might function as a rhogap by binding to Krit1.

SUGGESTIONS TO AUTHORS

The authours addressed the concerns from three reviewers except the following problems. These should be clarified before publication.

In the legend of Figure 6, explanation of (F) about the mutant is missing. What does mCcm2-stand for? Does m mean mutant? If m stands for mutant, wild type should be added to Ccm2. Is this Ccm(F217A)? The authors should thoroughly check the legends.

The authors use BEC for dual abbreviation; one is brain endothelial cell (Figure 4G) and brain endothelial cell in the text and reply to my question.

The authors should reanalyze the statistical significance by other methods.
They analyzed the significance by unpaired t-test even between two groups among more than three groups (Fig.2 G-J, Fig.5 G-K, Fig. 6I-K, and others in supplemental Figures). Statistical analyses among more than three groups should be done by ANOVA.

Intensity of RBC in the dorsal and posterior cardinal vein of cv29 is still weaker than WT in the replaced panels. These results still suggest the impairment of circulation of the trunk. This is consistent with Fig. 4H. Thus, to avoid misunderstandings, the authors describe the truth about Fig. 1F and 1H.

In the 4th setence of the result, what dos 'this' stand for? This stage is unclear.

Reviewer 2

I have no more comments.

Reviewer 3

SUMMARY OF THE ADVANCE MADE IN THIS PAPER AND ITS POTENTIAL SIGNIFICANCE TO THE FIELD

In this paper, Yang et al investigate the role of ARAP3 in the cerebral vasculature, using zebrafish as a model. This is a solid study indicating a relatively novel role for ARAP3 in the vasculature alongside a well-supported mechanism. It has been improved in review.

SUGGESTIONS TO AUTHORS

All my initial comments have been acted on, I have no further issues with this paper.

Second revision

Author response to reviewers' comments

© 2026. Published by The Company of Biologists under the terms of the Creative Commons Attribution License (https://creativecommons.org/licenses/by/4.0/).

Point by Point Response to the Reviewers

In the legend of Figure 6, explanation of (F) about the mutant is missing. What does mCcm2-stand for? Does m mean mutant? If m stands for mutant, wild type should be added to Ccm2. Is this Ccm(F217A)? The authors should thoroughly check the legends.

Response to this point: We thank the reviewer for identifying this lack of clarity. We apologize for the inconsistent nomenclature. "mCcm2" was intended to denote the F217A mutant of Ccm2. To avoid ambiguity, we have replaced "mCcm2" throughout the manuscript with "Ccm2(F217A)" and have designated the wild-type construct as "WT Ccm2". Where applicable, the constructs are now labeled as "WT Ccm2-FLAG" and "Ccm2(F217A)-FLAG". We have revised the legend for Fig. 6F accordingly and thoroughly checked all figure legends to ensure consistent nomenclature.

The authors use BEC for dual abbreviation; one is brain endothelial cell (Figure 4G) and brain endothelial cell in the text and reply to my question.

Response to this point: We thank the reviewer for identifying this inconsistency. We apologize for the incorrect and ambiguous description of BEC. We have corrected the inconsistent description and standardized "BECs" to "blood vascular endothelial cells" throughout the manuscript.

The authors should reanalyze the statistical significance by other methods.

They analyzed the significance by unpaired t-test even between two groups among more than three groups (Fig.2 G-J, Fig.5 G-K, Fig. 6I-K, and others in supplemental Figures). Statistical analyses among more than three groups should be done by ANOVA.

Response to this point: We thank the reviewer for this important suggestion. We have reanalyzed the data involving more than two groups in Fig. 2G-J, Fig. 5H-G, Fig. 6J-K, and the relevant supplementary figures using ANOVA followed by appropriate multiple-comparisons tests. For percentage data representing categorical outcomes in 5G, 5K and 6I, ANOVA is not appropriate. Therefore, we retained contingency-table analyses using chi-square tests. We have revised the statistical methods, figure legends, and significance annotations accordingly. For comparisons in which the revised analysis changed the statistical significance, we have updated the corresponding results and text in the manuscript.

Intensity of RBC in the dorsal and posterior cardinal vein of cv29 is still weaker than WT in the replaced panels. These results still suggest the impairment of circulation of the trunk. This is consistent with Fig. 4H. Thus, to avoid misunderstandings, the authors describe the truth about Fig. 1F and 1H.

Response to this point: We thank the reviewer for this important comment. The misleading image resulted from prolonged exposure to the anesthetic during image acquisition. We have replaced this image. After examining a large number of embryos, we did not observe impaired trunk circulation in the mutants at 48 hpf. We also clarify that the quantification shown in Fig. 1H was performed at 5 dpf, rather than at 48 hpf. This phenotype occurred later than the vascular malformation and hemorrhage phenotypes. We therefore consider it to be a secondary phenotype associated with pericardial edema, rather than evidence of a primary circulation defect in the trunk. To avoid misunderstanding, we have revised the text and figure legends to specify the observation and quantification time points and our interpretation of these findings.

In the 4th setence of the result, what dos 'this' stand for? This stage is unclear.

Response to this point: We thank the reviewer for pointing this out. In this context, "at this stage" was intended to mean "currently" rather than to refer to a specific developmental stage. To avoid ambiguity, we have replaced "at this stage" with "currently."

© 2026. Published by The Company of Biologists under the terms of the Creative Commons Attribution License (https://creativecommons.org/licenses/by/4.0/).

Third decision letter

MS ID#: dev.205762R2

MS TITLE: Loss of arap3 Drives a Cerebral Cavernous Malformation-Like Phenotype in Zebrafish via Krit1 Interaction and RhoA Dysregulation

AUTHORS: Di Yang, Zewen Tao, Si Chen, Hongcheng Wang, Lingfei Luo and Jingying Chen

Dear Dr Chen,

I am happy to tell you that your manuscript has been accepted for publication in Development, pending our standard publication integrity checks.

© 2026. Published by The Company of Biologists under the terms of the Creative Commons Attribution License (https://creativecommons.org/licenses/by/4.0/).
